# Social connections with family and friends in adolescence: Shaping body mass index trajectories into adulthood

**DOI:** 10.1016/j.ssmph.2025.101756

**Published:** 2025-01-16

**Authors:** Katie S. Taylor, Harry Tattan-Birch, Martin N. Danka, Liam Wright, Eleonora Iob, Daisy Fancourt, Yvonne Kelly

**Affiliations:** a Department of Epidemiology and Public Health, University College London, WC1E 7HB, London, UK; b Department of Behavioural Science and Health, University College London, WC1E 7HB, London, UK; c Centre for Longitudinal Studies, Social Research Institute, IOE, UCL's Faculty of Education and Society, University College London, London, UK

## Abstract

**Objectives:**

To investigate whether adolescent social connections influence body mass index (BMI) trajectories into adulthood and explore whether associations are moderated by gender, ethnicity or age.

**Methods:**

Data came from 17,719 American adolescents in grades 7–12 at baseline (1994–95) from the National Longitudinal Study of Adolescent to Adult Health. Growth curve models tested associations between baseline social connections and BMI trajectories from waves II-V including interactions for gender, ethnicity and age.

**Results:**

Stronger peer connections were associated with flatter BMI trajectories. For example, BMI for those with high peer contact was 0.79 kg/m^2^ lower [95% CI -1.20, −0.38] 22 years after baseline, compared to those with low contact. Stronger family connections were associated with steeper trajectories. For example, BMI for those with high family contact was 0.52 kg/m^2^ higher [95% CI 0.01, 1.02] 22 years after baseline, compared to those with low contact.

**Discussion:**

Among adolescents, stronger peer connections were associated with flatter BMI trajectories and stronger family connections with steeper trajectories. Promotion of peer-based interventions could be explored as a strategy to promote healthy weight trajectories.

## Highlights

• Adolescent social connections are associated with body mass index trajectories.
• Stronger peer social connections are associated with flatter adiposity trajectories.
• Stronger family connections are associated with steeper adiposity trajectories.
• No observable differences in associations were found by gender, ethnicity or age.

## 1 Introduction

In the past three decades, obesity prevalence has more than tripled among American adolescents (Hales et al.). Early life obesity can have significant immediate and longer-term impacts on health (Weihrauch-Blüher & Wiegand, 2018), highlighting the importance of identifying the determinants of obesity across the lifecourse.

Various psychosocial factors contributing to obesity have been explored, including social connections. Social connections are broadly defined as the ways in which individuals are connected emotionally, behaviourally, and physically to others. Social connections are commonly categorised into 3 dimensions (Holt- and Lunstad, 2018). First, structural aspects, which are typically quantitative, assess the number, diversity, or frequency of social connections. Second, functional aspects aim to collect the actual or perceived availability of resources that relationships may provide. Third, qualitative indicators assess the positive and negative aspects of relationships. Mechanistically, social connections can be linked to adiposity through either buffering (Cohen & Wills, 1985) or aggravating (Birmingham et al., 2018) psychological stress, via biological (e.g., the HPA-axis (McEwen, 1998)) and behavioural pathways (e.g., diet (Wardle & Gibson, 2002)). A comprehensive, policy-focussed, review of the associations between social relationships and both health behaviours and mental health has been conducted (Umberson et al., 2010a). A recent study also found that altered brain reactivity to food cues mediated associations between perceived social isolation and both maladaptive eating behaviours and increased body fat composition (Zhang et al., 2024).

Adolescence is a central life stage for both changes in social relationships and obesity development. Adolescence is characterised by rapid expansions of social networks and changing importance of different social relationships, as peers become increasingly influential and adolescents want more independence from their parents (Umberson, Crosnoe, & Reczek, 2010). This is in line with the social reorientation theory which posits that adolescent social interactions shift towards peers and away from caregivers (Nelson et al., 2016). Aligned with this theory, adolescents demonstrate an increased sensitivity to both peer rejection (Sebastian et al., 2010) and peer influence (Gardner & Steinberg, 2005). Although peer relationships are significant in adolescence, their unique effects are not consistently observed when compared to other social control conditions, such as interactions with family members (Cheng et al., 2024).

Furthermore, adolescence is considered a critical period for excessive weight gain (Alberga et al., 2012; Jasik & Lustig, 2008). A study, based in the US, showed that the prevalence of both overweight and obesity continued to rise between the ages of 9–19, concluding that puberty is a high-risk time for abnormal weight gain (Kimm et al., 2002). However, there is some conflicting evidence suggesting that obesity prevalence stabilises after age 11 (Howe et al., 2015). These increases in adiposity during adolescence may be due to physiological and behavioural changes. Potential physiological mechanisms include changes in the hormonal regulation of appetite, satiety and fat distribution that occur during puberty. For example, a normal increase in insulin secretion and resistance happens at puberty onset in girls and may facilitate the observed physiologic increase in adiposity (Jasik & Lustig, 2008). Changes in health behaviours also contribute to weight gain during adolescence. For example, a study showed a 26% decline in physical activity among 12-15-year-olds (Aaron et al., 2002) and the Growing Up Healthy Study showed an increase in fast food consumption between ages 9–12 and 13–14 which was associated with greater weight gain (Taveras et al., 2006). These behavioural changes may reflect the increased academic and social pressure experienced during adolescence. Considering this, it is essential that risk factors for obesity be addressed in adolescence to provide opportunities to change health trajectories.

Early life research has indicated links between social support, parent-child relationship quality, and peer relationship quality with adiposity. Empirical findings suggest that a lack of social support from parents has been linked with unhealthy weight control behaviours (Vander and Wal, 2012), higher BMI (Gerald et al., 1994), and increased waist-to-hip ratio over time (Midei & Matthews, 2009), among both children and adolescents. Links between parent-child relationship quality and obesity are less consistent. A meta-analysis found mostly small effect sizes for parent-child relations and child weight status and concluded a lack of support for making quality of parent-child relationships a main target for preventing obesity (Pinquart, 2014). Peer relationship quality has been explored in less detail, with findings suggesting that higher quality friendships are associated with reduced obesity risk in childhood (Boneberger et al., 2009) and adolescence (Sokol et al., 2019). Childhood obesity may also influence peer relationships, suggesting these associations are bidirectional (Sutter et al., 2020). However, as most studies exploring associations between social connections in early life and adiposity are cross-sectional in design, the direction of association is difficult to establish highlighting the need for more prospective longitudinal studies.

The relationship between social connections and adiposity may differ by gender, ethnicity, and age. There are gender differences in the contributors of obesity and social connections. A study observed that women who were single, widowed or divorced had higher odds of obesity whereas for men few associations were observed (Hosseini et al., 2020). These differences may be due to social relationships having different meanings for males and females. Another study observed differences in associations between social relationships and body weight by ethnicity and age. Specifically, White and African American participants with high emotional support had lower BMI (Lincoln & Nguyen, 2022) whilst Caribbean Black participants experienced the opposite. Furthermore, White participants aged 65+ and African American participants aged 50–64 with high emotional support had higher BMI, whereas the opposite for all other age groups was observed. Potential demographic differences have rarely been explored in adolescence.

In this study, we used data from The National Longitudinal Study of Adolescent to Adult Health (Add Health). The primary aim of this study was to investigate the associations between the social connection constructs (social contact, relationship status, perceived and received support, and positive and negative experiences) from family and friends in adolescence and BMI trajectories into adulthood from Waves II-V. The secondary aim was to investigate whether these associations were moderated by gender, ethnicity, or age. Five hypotheses were tested, with hypotheses 4 and 5 being exploratory due to lack of existing research:

1. Lower levels of social connections will be associated with greater increases in BMI over time, whilst higher levels of social connections will be associated with lower increases in BMI.
2. Associations will be stronger for peer-related compared to family-related social connections (Umberson, Crosnoe, & Reczek, 2010).
3. Associations will be stronger among females compared to males.
4. Associations will differ by ethnic group (Lincoln & Nguyen, 2022).
5. Associations will be stronger between peer connections and BMI among the older age group (Umberson, Crosnoe, & Reczek, 2010).

## 2 Methods

### 2.1 Dataset

The National Longitudinal Study of Adolescent to Adult Health (Add Health) is a school-based longitudinal study of a nationally representative sample of adolescents whom at baseline were in grades 7–12 in the United States in 1994–95 (Harris et al., 2019). For the in-home survey, adolescents were selected with unequal probability of selection from the 1994-95 school enrolment rosters from the identified schools. In total, 20,745 adolescents were recruited for the in-home interviews. Currently, 5 waves of data collection have been completed from 1994 to 2018 (wave I in 1994–95, wave II in 1996, wave III in 2001–02, wave IV in 2008–09, and wave V in 2016–18). For further information on timings, ages, response rates and dates, see supplementary index 1 (SI 1).

### 2.2 Sample

To be included in the analyses, participants needed to have data for social connections at baseline. They also needed to have data for BMI at baseline and at least one follow-up for BMI. Observations were dropped if the participant was currently pregnant at a given wave. This left an analytical sample of 17,719 participants (85% of recruited sample).

### 2.3 Measures

#### 2.3.1 Social connections

Social connections were measured at Wave I. Ten constructs were created based on either individual items or an index. A combination of two or more variables to create the constructs was primarily used, which were selected based on their theoretical and conceptual relevance to the underlying construct we aimed to measure, rather than relying on pre-existing scales. This was due to the study aiming to investigate different social connection constructs across the three dimensions which Add Health did not include composite measures on. We mapped the individual items onto their relevant constructs within each dimension based on Holt-Lunstad's definition of structural, functional and quality dimensions (Holt- and Lunstad, 2018). The structural constructs included social contact with family and friends, and relationship status. The functional constructs included loneliness, perceived support from family and friends, and received support from family and friends. The quality constructs included positive (e.g., satisfaction) and negative experiences (e.g., arguments) with family. When multiple items were used to create a construct, the item response options were collapsed to have the same number of responses as the item with the lowest number of response options. These items were then summed to create a total scale for that construct.

The 10 social connection constructs were categorised into high, moderate, and low levels as most responses were skewed. We decided to create these categories based on the qualitative meaning of the total numbers. For example, creating a ‘low’ perceived support group consisting of participants with a total score of 0–14 indicating that they answered 1 or 2 on a scale of 1–5 for 7 items, where 1 or 2 indicated lower support. SI 2 shows how constructs were created). An additional category of ‘no mum and/or dad’ was included for the family-related constructs, identified by the ‘not applicable’ response option to the family-related social connection measures.

#### 2.3.2 Adiposity

BMI, constructed from weight and height, was used as a measure of adiposity. At Wave I, height and weight were self-reported. At Waves II-V, height and weight were measured by a field examiner.

#### 2.3.3 Moderators

Gender, ethnicity, and age were included in analyses as moderators. Gender was categorised into male and female. Ethnicity was categorised into Hispanic, Non-Hispanic White, Non-Hispanic Black, and Non-Hispanic Asian. Age was measured at Wave I and categorised into 11-14-year-olds and 15-18-year-olds based on the timing of middle school.

#### 2.3.4 Covariates

The statistical analyses were adjusted for potential confounding variables, selected based on previous research in this field. These included: gender (Hosseini et al., 2020), ethnicity (Lincoln & Nguyen, 2022), childhood socioeconomic position (SEP) (parent education, parent occupation, household income, neighbourhood disadvantage) (Cameron et al., 2015; Weyers et al., 2008), self-rated health at baseline (Bauldry et al., 2012). As per previous research (Dennison & Swisher, 2019), a composite indicator of neighbourhood disadvantage was used based on the average of five census tract measures of socioeconomic disadvantage, including proportions of adults unemployed, families below poverty, households receiving public assistance, female-headed households, and respondents below 25 years without a bachelor's degree (Cronbach's *a* = 0.91). Health behaviours and psychological wellbeing are theorised to be mediators on the causal pathway from social connection to adiposity, so were not adjusted for (Umberson et al., 2010a).

### 2.4 Statistical analyses

The longitudinal associations of social connection constructs with BMI were estimated using linear growth curve models. This method is well-suited for analysing longitudinal data as both within-subject changes over time and between-subject variations can be examined. As data collection timepoints were not evenly spaced (e.g., one year between waves I and II versus seven years between waves III and IV), the wave variable was transformed into ‘years since baseline’ to provide an even measurement of time. In the current study, linear models were selected over quadratic models as they proved a better fit for the data based on the Akaike Information Criteria, Bayesian Information Criteria and likelihood ratio test. Interactions between time and the social connection constructs were included to allow trajectory estimations for each level of social connection (low, moderate, high). To establish a clear temporal sequence between social connections and adiposity, BMI at baseline was adjusted for in analyses. Ten individual models were run for each social connection construct in the main analyses. To explore the differences by gender, ethnicity, and age, three-way interaction effects were tested. Cross-sectional survey weights for Wave I were included in the analyses to account for the oversampling of certain groups (e.g., black adolescents with college-educated parents). Clustering by school was accounted for by including school identification in the models. P-values were corrected for multiple testing using the Benjamini-Hochberg procedure. Average marginal effects based on the growth curve model output were calculated, reported, and graphically depicted to improve the interpretability of the results.

Missing data were estimated using multiple imputation by chained equations (Van et al., 2020). In the analytical sample, there were 4121 missing observations for BMI at wave II, 4326 at wave III, 3347 at wave IV and 12758 at wave V. Imputations were completed in wide format and transformed into long format for analysis; this allowed us to use prior observations of BMI to impute later values (see SI 3 for between wave BMI correlations). Twenty imputed datasets with 3 iterations were created based on percentage of missing data and estimates were pooled using Rubin's Rule. Interactions between social connection constructs and gender, ethnicity or age were included in the imputation model to best reflect the analytical model. Data management and multiple imputation analyses were performed in R 4.1.2 using the R packages *tidyverse* (Wickham et al., 2019) and *mice* (Van et al., 2000), respectively. Regression analyses were conducted in R using the package *lme4* (Bates, Mächler, Bolker, & Walker, 2015) and marginal effects were calculated using the *marginaleffects* package (Arel-Bundock et al.).

#### 2.4.1 Supplemental and sensitivity analyses

First, we ran models that did not adjust for any covariates. Second, we ran models that did not adjust for baseline BMI to ensure that the models were not over adjusted. Third, we included depression as an additional covariate; this was not included in the main analyses as it could be an intermediate variable on the causal pathway leading from social connections to adiposity. Fourth, we included whether participants had reached puberty at baseline as an additional covariate as puberty may influence both social connections and BMI. Finally, we ran models that adjusted for all other social connection variables to understand whether each social connection construct is associated with BMI trajectories independent of each other.

### 2.5 Pre-registration

The protocol for the current study was pre-registered on Open Science Framework in January 2023 (DOI: https://doi.org/10.17605/OSF.IO/3EQPX). Originally, we planned to look at both BMI and waist circumference as outcomes. However, after assessing the data, we decided not to include waist circumference as it was only measured at two timepoints (Wave IV and V) and had large amounts of missing data. We had originally intended to include Caribbean Black as a subgroup of ethnicity, but parent's country of origin was not available at baseline, so this could not be completed. We also originally planned to include Native American as a subgroup, but the sample size (N = 95) was deemed too small. We also adapted the way that race and ethnicity were categorised to be in line with previous Add Health studies. We initially intended to include social network size as a structural construct in our analyses. However, upon closer inspection of the codebooks, we realised that the available items were not suitable for this purpose. Specifically, our plan was to include an item that asked participants to nominate up to 5 male and 5 female friends to generate a social network size score. However, this item was administered to only 7106 participants, while the majority were asked to nominate just 1 male friend and 1 female friend. Including this measure would have substantially reduced our sample size. We also considered incorporating a measure of social participation, but the relevant item primarily captured sports participation, which could be considered as a proxy for exercise rather than broader social engagement.

## 3 Results

### 3.1 Descriptive statistics

The final sample includes 17,719 participants. Table 1 displays the sample characteristics. The average age of the sample was ∼15.55 (SD ± 1.67) with 49.1% male. 55.2% of the sample was Non-Hispanic White (n = 9775), 21.4% Non-Hispanic Black (n = 3785), 17.1% Hispanic (n = 3026), and 6.4% Non-Hispanic Asian (n = 1133). SI 4 shows the distribution of BMI across the five waves. The mean and standard deviation of BMI increased over time. The percentages of participants with each social connection construct level at Wave I are outlined in SI 5 and the frequencies of participants with each BMI category classification from waves I-V can be found in SI 6.

**Table 1:** Sample characteristics for the imputed analytical sample, non-imputed analytical sample, and recruited sample.

	Imputed analytical sample (n = 17,719)	Non-imputed analytical sample (n = 17,719)	Recruited sample (n = 20,745)
**Age**			
Mean (SD)	15.545 (1.668)	15.545 (1.668)	15.657 (1.746)
Range	11.000–18.000	11.000–18.000	11.000–21.000
**Gender**			
Male	8705 (49.1%)	8705 (49.1%)	10263 (49.5%)
Female	9014 (50.9%)	9014 (50.9%)	10482 (50.5%)
**Ethnicity**			
Non-Hispanic White	9775 (55.2%)	9757 (55.5%)	11034 (53.7%)
Hispanic	3026 (17.1%)	2917 (16.6%)	3525 (17.1%)
Non-Hispanic Asian	1133 (6.4%)	1129 (6.4%)	1331 (6.5%)
Non-Hispanic Black	3785 (21.4%)	3775 (21.5%)	4479 (21.8%)
**Household income**			
Mean (SD)	45.706 (52.170)	46.481 (51.352)	45.728 (51.617)
Range	0.000–999.000	0.000–999.000	0.000–999.000
**Mother education level**			
Less than high school	3158 (17.8%)	2604 (17.1%)	3183 (18.2%)
Graduated high school	5111 (28.8%)	4438 (29.2%)	5127 (29.3%)
Trade school/some college	5243 (29.6%)	4545 (29.9%)	5190 (29.6%)
College +	4206 (23.7%)	3621 (23.8%)	4027 (23.0%)
**Mother occupation**			
No mother	977 (5.5%)	974 (5.5%)	1257 (6.1%)
Non-manual	8655 (48.8%)	8633 (48.9%)	9795 (47.4%)
Manual	2913 (16.4%)	2902 (16.4%)	3446 (16.7%)
Other	2680 (15.1%)	2673 (15.1%)	3135 (15.2%)
Unemployed	2479 (14.1%)	2483 (14.1%)	3017 (14.6%)
**Neighbourhood disadvantage**			
Mean (SD)	15.339 (6.612)	15.259 (6.550)	15.365 (6.623)
Range	3.871–57.279	3.871–57.279	3.871–59.800
**Self-rated health**			
Excellent	5012 (28.3%)	5012 (28.3%)	5835 (28.2%)
Very good	7064 (39.9%)	7063 (39.9%)	8096 (39.1%)
Good	4429 (25.0%)	4429 (25.0%)	5307 (25.6%)
Fair/Poor	1214 (6.9%)	1214 (6.9%)	1481 (7.1%)

### 3.2 Growth trajectories by level of social connection

**Fig. 1:**
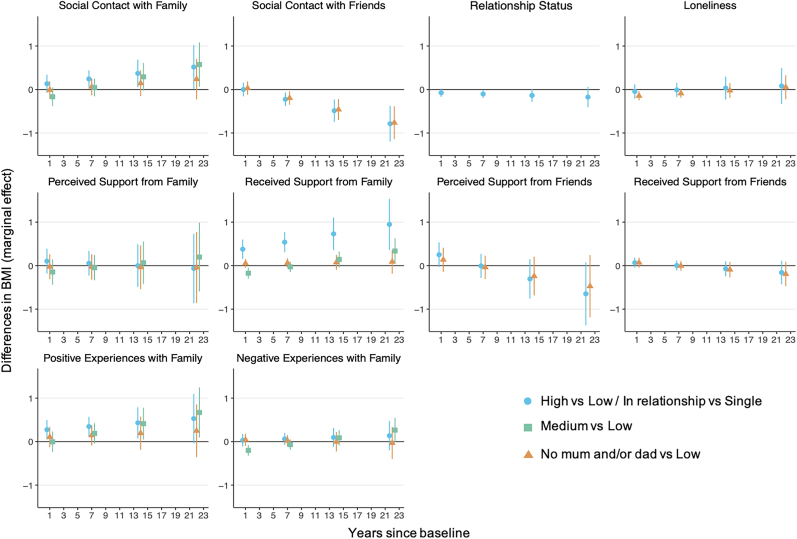
Marginal effects for BMI by social connections from growth curve models.

The longitudinal associations of social connection constructs with BMI were estimated using linear growth curve models. Fig. 1 shows marginal effects from the linear BMI growth curve models by level of social connection constructs (see SI 7 for multilevel output). In our baseline model, BMI at Wave II was similar across the levels of social connections, but the rate of BMI change over time differed by level of social contact with family and friends, received support from family, perceived support from friends and positive aspects with family. There was little difference in rate of BMI change by levels of relationship status, loneliness, received support from friends, perceived support from family, or negative aspects with family.

For the peer-related constructs, the rate of BMI increase was slower for those with higher contact or support compared to those with low contact or support. For example, the BMI of those with high social contact with friends was 0.49 kg/m^2^ lower [95% CI -0.74, −0.23] 14 years after baseline and 0.79 kg/m^2^ lower [95% CI -1.20, −0.38] 22 years after baseline, compared to those with low social contact.

For the family-related constructs, the rate of BMI increase was steeper for those with higher levels of contact or support, compared to those with low contact or support. The BMI of those with high social contact with family was 0.24 kg/m^2^ higher [95% CI 0.04, 0.44] 7 years after baseline and 0.52 kg/m^2^ higher [95% CI 0.01, 1.02] 22 years after baseline, compared to those with low social contact. The BMI of those with high received support from family was 0.54 kg/m^2^ higher [95% CI 0.31, 0.77] 7 years after baseline and 0.95 kg/m^2^ higher [95% CI 0.36, 1.54] 22 years after baseline, compared to those with low received support. The BMI of those with high positive aspects with family was 0.35 kg/m^2^ higher [95% CI 0.13, 0.57] 7 years after baseline and 0.43 kg/m^2^ higher [95% CI 0.08, 0.79] 14 years after baseline, compared to those with low positive aspects. The difference in BMI between high and low social connection groups continues to increase between 6 and 12 years after baseline.

### 3.3 Growth trajectories by level of social connection and gender, age, or ethnicity

Using three-way interactions, no detectable differences in associations between social connections and BMI over time by gender, ethnicity or age were observed (SI 8, 9, 10). However, as estimates may be imprecise (e.g., as shown by the wide confidence intervals on the three-way interactions), we cannot rule out there being any differences.

### 3.4 Sensitivity analyses

A similar pattern of results was observed for models that did not adjust for covariates or baseline BMI and models that did adjust for depression, timing of puberty and other social connection constructs (see SI 11, 12, 13, 14, 15).

## 4 Discussion

The current study explored the associations between family and peer social connection dimensions in adolescence with BMI trajectories into adulthood. Findings suggest that stronger family connections may be associated with steeper increases in BMI over time, whilst stronger peer connections may be associated with flatter increases in BMI over time.

The finding that stronger family connections may be associated with higher BMI trajectories does not support our first hypothesis of protective influences of any social connections. Existing research typically observes protective effects of early life social connections with parents on adiposity or null effects. Regarding social support, higher parental support has been associated with lower adolescent BMI (Yayan & Çelebioğlu, 2018) whilst a lack of parental support has been linked with unhealthy weight control behaviours (Vander and Wal, 2012), higher BMI (Gerald et al., 1994), and increased waist-to-hip ratio in early life (Midei & Matthews, 2009). Contrastingly, our findings suggest that received parental social support was associated with increases in BMI overtime. This finding may be due to the measure of received support reflecting other factors associated with BMI. For example, participants that have not spoken to their parents about a personal problem are not necessarily less supported, they may have just not experienced any personal problems to discuss. In fact, it may be that those discussing personal problems with their parents have increased psychological stress contributing to weight gain (Milam et al., 2017). Regarding the quality of parental social relationships, there are mixed findings for the associations with adiposity. For example, higher levels of parental warmth have been associated with decreasing/stable child BMI during a family-based behavioural weight control programme (Rhee et al., 2016) yet indulgent parenting (high nurturance and low control) has been associated with higher obesity risk (Olvera & Power, 2010; Wake et al., 2007). The current findings are more in line with the latter as more positive experiences with family were associated with higher BMI trajectories. More positive experiences could be characteristic of an indulgent parenting style which could lead to more lenient attitudes towards health behaviours. The behavioural and psychological mechanisms of these relationships should be investigated further, including the family food environment.

The finding that stronger peer connections may be associated with lower BMI over time supports both our first and second hypotheses. Among adolescents, higher quality friendships have been shown to be protective against higher average excess BMI (Sokol et al., 2019), associations that may be bidirectional (Sutter et al., 2020). Our findings are in line with the existing research by suggesting that higher social contact with friends and perceived support from friends may be associated with flatter increases in BMI over time. These findings align with the social reorientation theory that states in adolescence there is a shift from caregivers to peers having more influence on behaviours (Nelson et al., 2016; Umberson, Crosnoe, & Reczek, 2010). Recently, however, the unique influence of peers in adolescence has been questioned (Cheng et al., 2024). Nonetheless, exposure to positive peer relationships may improve adolescent resilience (Shao & Kang, 2022) and self-esteem (Gruenenfelder-Steiger et al., 2016). These factors may be related to reduced stress levels (Schraml et al., 2011) and thus limit the behavioural and biological impact of stress on BMI.

The present study has several strengths. The longitudinal design means we could account for the possibility of reverse causation. The large sample size and oversampling of underrepresented groups improves statistical power, precision and generalisability. Comparing across social connection dimensions and sources can enable the development of targeted interventions. However, there are also several limitations. Whilst we have attempted to include measured confounders, unmeasured and poorly measured confounders remain. Whilst the overall sample size was large, the subgroup analyses likely lacked power to detect effects, indicated by the wide confidence intervals. The measures of some social connection variables may not accurately reflect the constructs they are proposed to measure (i.e., measurement error). Using BMI as a measure of adiposity is limited as it cannot distinguish fat mass from muscle mass and provides no information on fat distribution. Future research should prioritise the use of more robust measures of social connections and more objective measures of adiposity to explore these relationships in greater detail. Although the associations between social connections and BMI trajectories were statistically significant, the changes in BMI were small (e.g., 0.25 percentage points), suggesting limited clinical significance. This distinction is important because statistically significant findings may not always result in meaningful health outcomes or warrant changes in clinical practice.

To conclude, the current findings suggest that, during adolescence, stronger peer social connections may contribute to flatter BMI trajectories, whilst stronger family social connections may contribute to steeper BMI trajectories. This suggests that it is necessary to consider the complex nature of social relationships in adolescence when addressing health outcomes.

## CRediT authorship contribution statement

**Katie S. Taylor:** Writing – review & editing, Writing – original draft, Visualization, Methodology, Investigation, Formal analysis, Data curation, Conceptualization. **Harry Tattan-Birch:** Writing – review & editing, Visualization, Supervision, Methodology, Formal analysis. **Martin N. Danka:** Writing – review & editing, Visualization, Methodology, Formal analysis. **Liam Wright:** Writing – review & editing, Visualization, Supervision, Methodology, Formal analysis. **Eleonora Iob:** Writing – review & editing, Supervision, Methodology, Investigation, Formal analysis, Conceptualization. **Daisy Fancourt:** Writing – review & editing, Supervision, Methodology, Formal analysis, Conceptualization. **Yvonne Kelly:** Writing – review & editing, Supervision, Methodology, Investigation, Formal analysis, Conceptualization.

## Public health implications

The current findings suggest that peer relationships may play a protective role in promoting healthier weight trajectories. Peer-based interventions, such as school-based programmes, could be explored to assess if they can causally contribute to reducing obesity. The family-related findings also suggest that certain family dynamics or health behaviours within the household could unintentionally promote unhealthy habits, which could have implications for positive parenting practices. However, it will be important in future experimental studies to explore the effect size achievable and, therefore, how such interventions compare to other obesity prevention strategies.

## Ethical statement

This study used data from The Longitudinal Study of Adolescent to Adult Health (Add Health) study. All procedures performed in studies involving human participants were in accordance with the ethical standards of the institutional and/or national research committee and with the 1964 Helsinki declaration and its later amendments or comparable ethical standards.

Add Health participants provided written informed consent for participation in all aspects of Add Health in accordance with the University of North Carolina School of Public Health Institutional Review Board guidelines that are based on the Code of Federal Regulations on the Protection of Human Subjects 45CFR46: https://www.hhs.gov/ohrp/humansubjects/guidance/45cfr46.html.

## Declaration of competing interest

The authors declare that they have no known competing financial interests or personal relationships that could have appeared to influence the work reported in this paper.

## Data Availability

The authors do not have permission to share data.
