# Supplementary material for: Social connections with family and friends in adolescence: Shaping body mass index trajectories into adulthood
Source: SSM Popul Health. 2025 Jan 16;29:101756. doi: 10.1016/j.ssmph.2025.101756 (PMC11787614; doi:10.1016/j.ssmph.2025.101756)
Supplement: Multimedia component 1 [file mmc1.docx]

# Supplementary Index

##
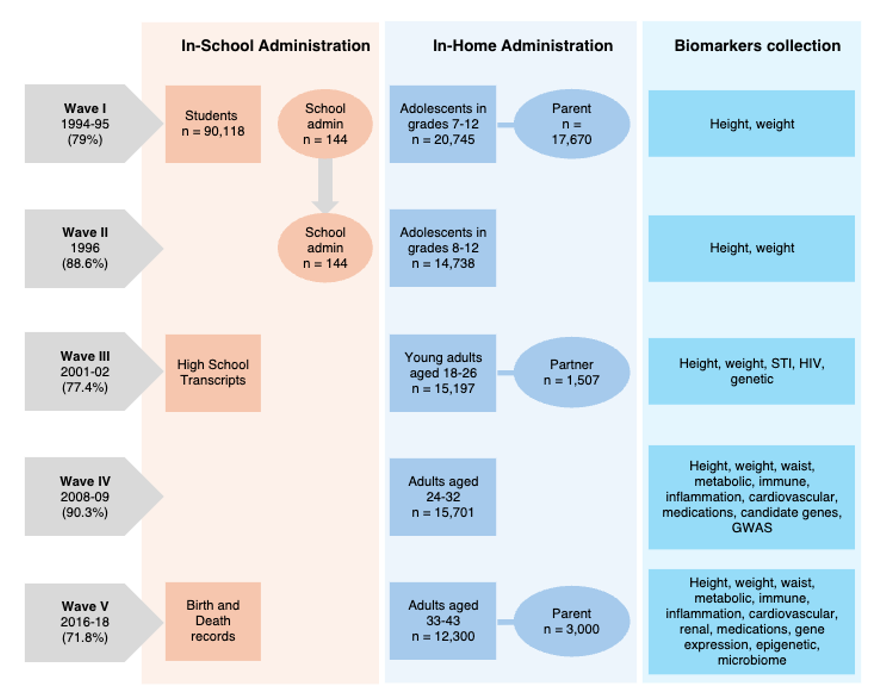
Supplementary index 1: Details on the Add Health study design

*Note.* This is a figure adapted from the Add Health website which includes the dates of data collection, response rates for each wave, and ages of participants at each wave.

## Supplementary index 2: Items used to create the social connection constructs.

| **Dimension** | **Construct** | **Items used to create construct** | **How construct was created** |
| --- | --- | --- | --- |
| **Structural** | Family social contact | How often is your mother at home when you leave for school? (1)  How often is your mother at home when you return from school? (2)  How often is your mother at home when you go to bed? (3)  How often is your father at home when you leave for school? (4)  How often is your father at home when you return from school? (5)  How often is your father at home when you go to bed? (6)  On how many of the past 7 days was at least 1 parent in the room with you while you ate dinner? (7) | Scales were adapted to have the number of responses equal to the item with the lowest number of responses (5 response options). Most variables were reverse coded (1-6). All response options were then added to create a total social contact measure ranging from 0-28. This was then split into low (0-13), medium (14-20), and high (21-28) based on meaning of the response options. |
|  | Friend social contact | In the past week, how many times did you hang out with friends? | Scale was changed from 0 (not at all), 1 (1 or 2 times), 2 (3 or 4 times) and 3 (5+ times) to low (0), medium (1, 2) and high (3). |
|  | Relationship status | Are any of your nominated romantic relationships still ongoing? | If respondent answered yes, they were classified as in a relationship (1), if ‘no’ then classified as single (0). |
| **Functional** | Loneliness | How often in the past week did you feel lonely? | Scale was changed from 0 (never or rarely), 1 (sometimes), 2 (a lot), 3 (most or all of the time) to low (0), medium (1), high (2, 3). |
|  | Family perceived support | How much do you think your resident mum cares about you?  How much do you think your resident dad cares about you?  How much do you feel your family pays attention to you?  How much do you feel your family understands you? | Responses were added together to create a total perceived support from family measure, ranging from 0-16. This was then split into low (0-8), medium (9-12) and high (13-16) based on meaning of responses. |
|  | Friend perceived support | How much do you feel your friends care about you? | Scale was changed from 1 (not at all), 2 (very little), 3 (somewhat), 4 (quite a bit), and 5 (very much) to low (1, 2), medium (3, 4), and high (5) based on meaning of responses. |
|  | Family received support | In the past 4 weeks, have you spoken to your mum about a personal problem?  In the past 4 weeks, have you worked on a school project with your mum?  In the past 4 weeks, have you spoken to your dad about a personal problem?  In the past 4 weeks, have you worked on a school project with your dad? | Responses were added together to create a total received support from family measure, ranging from 0-4. This was then split into low (0), medium (1, 2) and high (3, 4) based on meaning of responses. |
|  | Friend received support | In the past 4 weeks, have you spoken to a male friend about a problem?  In the past 4 weeks, have you spoken to a female friend about a problem? | Responses were added together to create a total received support friends measure, ranging from 0-2. This was then split into low (0), medium (1) and high (2) based on meaning of responses. |
| **Quality** | Positive | Most of the time your mum is warm and loving towards you. (1)  Overall, you are satisfied with the relationship with your mum. (2)  Most of the time your dad is warm and loving towards you. (3)  Overall, you are satisfied with the relationship with your dad. (4)  How close do you feel to your resident mum? (5)  How close do you feel to your resident dad? (6) | Some scales (1-4) were reverse coded from 1 (strongly agree), 2 (agree), 3 (neither), 4 (disagree), 5 (strongly disagree). All item responses were then summed to create a total positive aspects measure, with a minimum of 0 and a maximum of 24. These were then split into low (0-12), medium (13-18) and high (19-24) based on patterns in the data and frequencies. |
|  | Negative | In the past week, have your argued with your mum?  In the past week, have you argued with your dad? | Responses were added together to create a total negative aspects measure with a minimum of 0 and a maximum of 2. These were then split into no negative (0), medium negative (1) or high negative (2) based on meaning of the responses. |

## Supplementary index 3: Correlation coefficients between BMI at each wave.


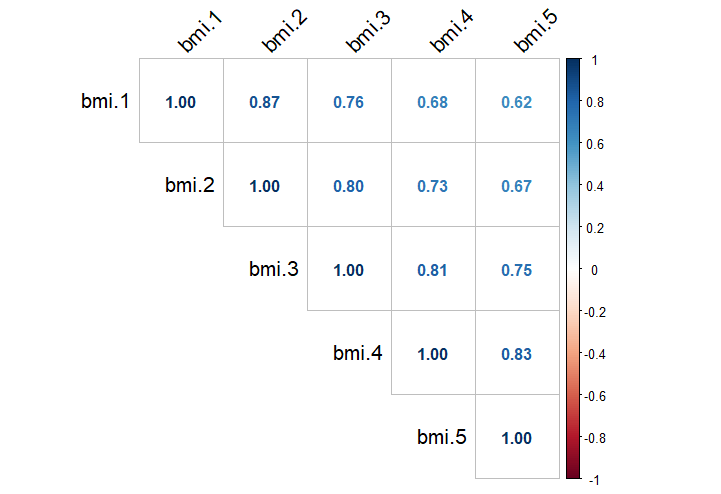


##
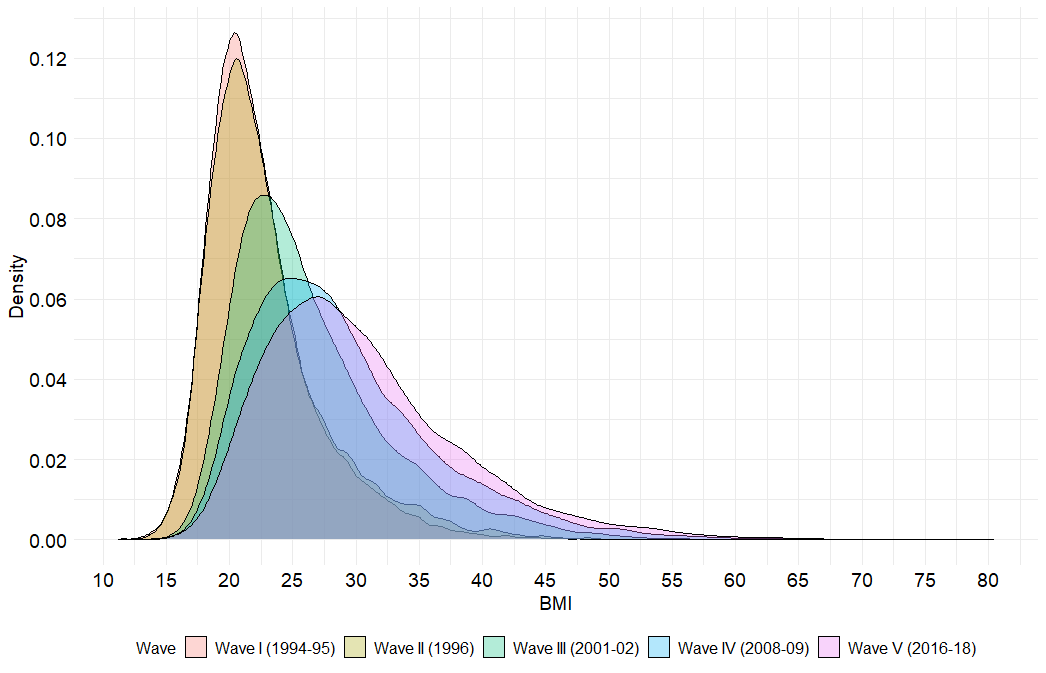
Supplementary index 4: Histograms of body mass index from Wave I to Wave IV in the Add Health sample.

##
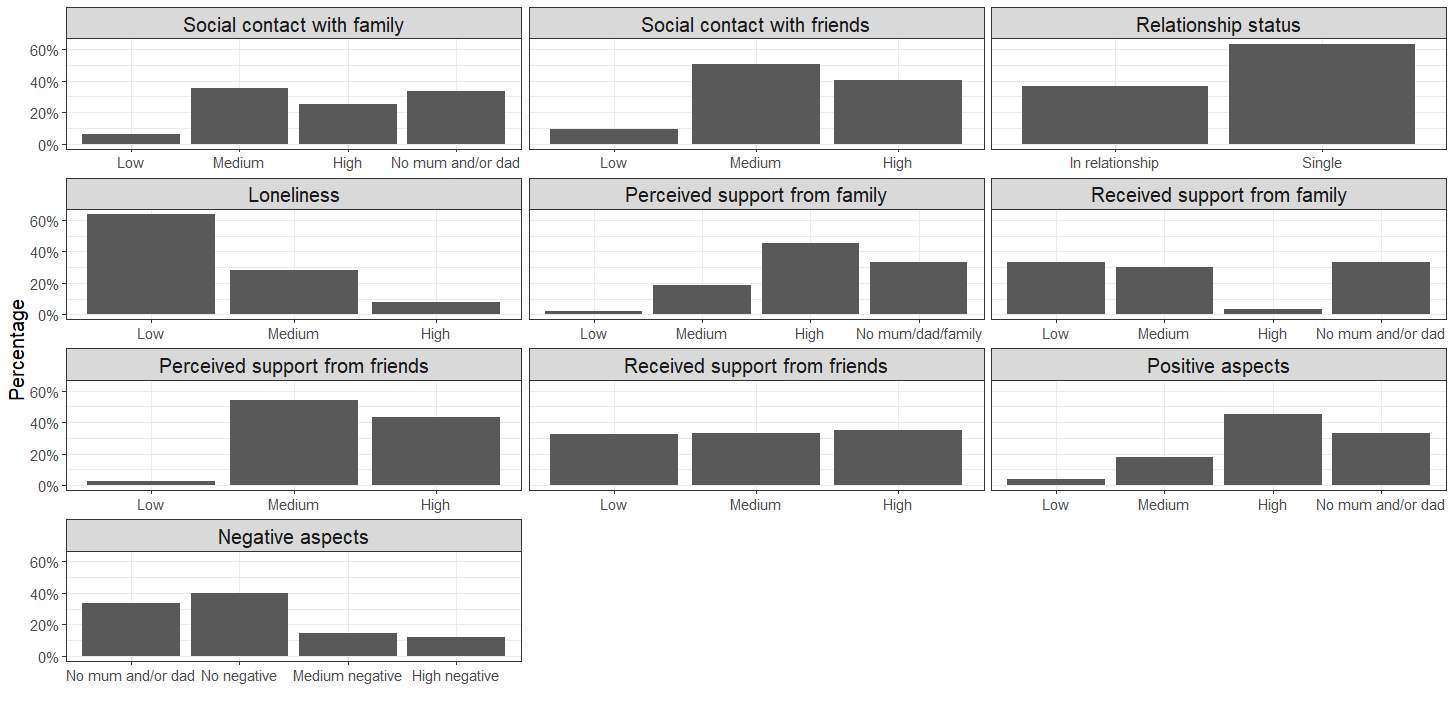
Supplementary index 5: Social connection construct percentages.

## Supplementary index 6: Frequencies and percentages of participants with each BMI category from waves I-V.

| **BMI category** | **N (%)** |
| --- | --- |
| **Wave I** |  |
| Underweight | 2399 (13.5%) |
| Normal weight | 11333 (64.0%) |
| Overweight | 2726 (15.4%) |
| Obesity | 1261 (7.1%) |
| **Wave II** |  |
| Missing | 4121 |
| Underweight | 1766 (13.0%) |
| Normal weight | 8432 (62.0%) |
| Overweight | 2134 (15.7%) |
| Obesity | 1266 (9.3%) |
| **Wave III** |  |
| Missing | 4326 |
| Underweight | 375 (2.8%) |
| Normal weight | 6131 (45.8%) |
| Overweight | 3739 (27.9%) |
| Obesity | 3148 (23.5%) |
| **Wave IV** |  |
| Missing | 3347 |
| Underweight | 201 (1.4%) |
| Normal weight | 4492 (31.3%) |
| Overweight | 4391 (30.6%) |
| Obesity | 5288 (36.8%) |
| **Wave V** |  |
| Missing | 12758 |
| Underweight | 38 (0.8%) |
| Normal weight | 1205 (24.3%) |
| Overweight | 1448 (29.2%) |
| Obesity | 2270 (45.8%) |

## Supplementary index 7: Results for growth curve models.

| **Term** | **Estimate** | **Std error** | **P value** | **2.5 %** | **97.5 %** | **FDR P value** |
| --- | --- | --- | --- | --- | --- | --- |
| **Social contact with family** |  |  |  |  |  |  |
| Intercept | 2.36 | 0.32 | 0.00 | 1.72 | 3.00 | 0.00 |
| Year | 0.33 | 0.01 | 0.00 | 0.30 | 0.35 | 0.00 |
| Social contact (medium) | -0.02 | 0.11 | 0.84 | -0.23 | 0.19 | 0.85 |
| Social contact (high) | 0.11 | 0.11 | 0.31 | -0.11 | 0.34 | 0.50 |
| Social contact (no mum and/or dad) | -0.20 | 0.12 | 0.08 | -0.43 | 0.03 | 0.16 |
| Year*Social contact (medium) | 0.01 | 0.01 | 0.34 | -0.01 | 0.04 | 0.53 |
| Year*Social contact (high) | 0.02 | 0.01 | 0.18 | -0.01 | 0.05 | 0.31 |
| Year*Social contact (no mum and/or dad) | 0.04 | 0.01 | 0.01 | 0.01 | 0.06 | 0.04 |
| **Social contact with friends** |  |  |  |  |  |  |
| Intercept | 2.25 | 0.28 | 0.00 | 1.70 | 2.79 | 0.00 |
| Year | 0.38 | 0.01 | 0.00 | 0.36 | 0.40 | 0.00 |
| Social contact (medium) | 0.07 | 0.08 | 0.40 | -0.09 | 0.23 | 0.57 |
| Social contact (high) | 0.04 | 0.08 | 0.65 | -0.13 | 0.20 | 0.73 |
| Year*Social contact (medium) | -0.04 | 0.01 | 0.00 | -0.06 | -0.02 | 0.00 |
| Year*Social contact (high) | -0.04 | 0.01 | 0.00 | -0.06 | -0.02 | 0.00 |
| **Relationship status** |  |  |  |  |  |  |
| Intercept | 2.43 | 0.29 | 0.00 | 1.85 | 3.01 | 0.00 |
| Year | 0.35 | 0.01 | 0.00 | 0.34 | 0.36 | 0.00 |
| Relationship status (single) | -0.07 | 0.05 | 0.18 | -0.17 | 0.03 | 0.31 |
| Year*Relationship status (single) | 0.00 | 0.01 | 0.45 | -0.02 | 0.01 | 0.59 |
| **Loneliness** |  |  |  |  |  |  |
| Intercept | 2.27 | 0.30 | 0.00 | 1.69 | 2.86 | 0.00 |
| Year | 0.35 | 0.00 | 0.00 | 0.34 | 0.36 | 0.00 |
| Loneliness (medium) | -0.15 | 0.05 | 0.01 | -0.26 | -0.05 | 0.02 |
| Loneliness (high) | -0.05 | 0.09 | 0.56 | -0.22 | 0.12 | 0.69 |
| Year*Loneliness (medium) | 0.01 | 0.01 | 0.20 | -0.01 | 0.02 | 0.34 |
| Year*Loneliness (high) | 0.01 | 0.01 | 0.58 | -0.02 | 0.03 | 0.69 |
| **Perceived support from family** |  |  |  |  |  |  |
| Intercept | 2.37 | 0.34 | 0.00 | 1.69 | 3.04 | 0.00 |
| Year | 0.35 | 0.02 | 0.00 | 0.31 | 0.39 | 0.00 |
| Perceived support (medium) | -0.02 | 0.16 | 0.87 | -0.33 | 0.28 | 0.88 |
| Perceived support (high) | 0.11 | 0.16 | 0.48 | -0.20 | 0.42 | 0.61 |
| Perceived support (no mum and/or dad) | -0.16 | 0.16 | 0.31 | -0.47 | 0.15 | 0.50 |
| Year*Perceived support (medium) | 0.00 | 0.02 | 0.97 | -0.04 | 0.04 | 0.97 |
| Year*Perceived support (high) | -0.01 | 0.02 | 0.71 | -0.05 | 0.03 | 0.75 |
| Year*Perceived support (no mum and/or dad) | 0.02 | 0.02 | 0.44 | -0.03 | 0.06 | 0.58 |
| **Received support from family** |  |  |  |  |  |  |
| Intercept | 2.43 | 0.31 | 0.00 | 1.82 | 3.04 | 0.00 |
| Year | 0.34 | 0.01 | 0.00 | 0.33 | 0.35 | 0.00 |
| Received support (medium) | 0.05 | 0.06 | 0.38 | -0.06 | 0.16 | 0.57 |
| Received support (high) | 0.35 | 0.12 | 0.00 | 0.12 | 0.59 | 0.01 |
| Received support (no mum and/or dad) | -0.20 | 0.07 | 0.00 | -0.33 | -0.06 | 0.01 |
| Year*Received support (medium) | 0.00 | 0.01 | 0.79 | -0.01 | 0.02 | 0.81 |
| Year*Received support (high) | 0.03 | 0.02 | 0.08 | 0.00 | 0.06 | 0.14 |
| Year*Received support (no mum and/or dad) | 0.02 | 0.01 | 0.00 | 0.01 | 0.04 | 0.01 |
| **Perceived support from friends** |  |  |  |  |  |  |
| Intercept | 2.08 | 0.32 | 0.00 | 1.45 | 2.71 | 0.00 |
| Year | 0.38 | 0.02 | 0.00 | 0.35 | 0.42 | 0.00 |
| Perceived support (medium) | 0.16 | 0.15 | 0.28 | -0.13 | 0.46 | 0.46 |
| Perceived support (high) | 0.29 | 0.15 | 0.05 | -0.01 | 0.59 | 0.11 |
| Year*Perceived support (medium) | -0.03 | 0.02 | 0.13 | -0.07 | 0.01 | 0.23 |
| Year*Perceived support (high) | -0.04 | 0.02 | 0.03 | -0.08 | 0.00 | 0.07 |
| **Received support from friends** |  |  |  |  |  |  |
| Intercept | 2.28 | 0.29 | 0.00 | 1.70 | 2.85 | 0.00 |
| Year | 0.36 | 0.01 | 0.00 | 0.35 | 0.37 | 0.00 |
| Received support (medium) | 0.08 | 0.06 | 0.18 | -0.04 | 0.20 | 0.31 |
| Received support (high) | 0.08 | 0.06 | 0.19 | -0.04 | 0.20 | 0.32 |
| Year*Received support (medium) | -0.01 | 0.01 | 0.09 | -0.03 | 0.00 | 0.17 |
| Year*Received support (high) | -0.01 | 0.01 | 0.13 | -0.02 | 0.00 | 0.23 |
| **Positive experiences with family** |  |  |  |  |  |  |
| Intercept | 2.17 | 0.33 | 0.00 | 1.51 | 2.83 | 0.00 |
| Year | 0.33 | 0.01 | 0.00 | 0.30 | 0.36 | 0.00 |
| Positive (medium) | 0.10 | 0.13 | 0.43 | -0.15 | 0.35 | 0.58 |
| Positive (high) | 0.26 | 0.12 | 0.03 | 0.03 | 0.50 | 0.07 |
| Positive (no mum and/or dad) | -0.03 | 0.13 | 0.78 | -0.28 | 0.21 | 0.81 |
| Year*Positive (medium) | 0.01 | 0.02 | 0.67 | -0.02 | 0.04 | 0.74 |
| Year*Positive (high) | 0.01 | 0.01 | 0.42 | -0.02 | 0.04 | 0.57 |
| Year*Positive (no mum and/or dad) | 0.03 | 0.02 | 0.03 | 0.00 | 0.06 | 0.08 |
| **Negative experiences with family** |  |  |  |  |  |  |
| Intercept | 2.47 | 0.31 | 0.00 | 1.86 | 3.08 | 0.00 |
| Year | 0.34 | 0.01 | 0.00 | 0.33 | 0.35 | 0.00 |
| Negative (medium) | 0.05 | 0.08 | 0.54 | -0.10 | 0.20 | 0.68 |
| Negative (high) | 0.03 | 0.08 | 0.73 | -0.13 | 0.18 | 0.76 |
| Negative (no mum and/or dad) | -0.22 | 0.07 | 0.00 | -0.35 | -0.09 | 0.00 |
| Year*Negative (medium) | 0.00 | 0.01 | 0.73 | -0.02 | 0.02 | 0.76 |
| Year*Negative (high) | 0.01 | 0.01 | 0.57 | -0.01 | 0.02 | 0.69 |
| Year*Negative (no mum and/or dad) | 0.02 | 0.01 | 0.00 | 0.01 | 0.04 | 0.01 |

Note. This table represents findings from linear mixed effect models controlling for age, gender, ethnicity, baseline BMI, neighbourhood disadvantage, parental education and occupation, and self-rated health. *FDR stands for false discovery rate adjustment, using the Benjamini Hochberg procedure.

## Supplementary index 8: Results for growth curve model by gender.

| **Term** | **Estimate** | **Std error** | **P value** | **2.5 %** | **97.5 %** | **FDR P value** |
| --- | --- | --- | --- | --- | --- | --- |
| **Social contact with family** |  |  |  |  |  |  |
| Intercept | 2.47 | 0.33 | 0.00 | 1.81 | 3.13 | 0.00 |
| Year | 0.33 | 0.02 | 0.00 | 0.29 | 0.37 | 0.00 |
| Gender (female) | 0.12 | 0.20 | 0.54 | -0.27 | 0.52 | 0.76 |
| Social contact (medium) | -0.02 | 0.16 | 0.90 | -0.33 | 0.29 | 0.94 |
| Social contact (high) | 0.06 | 0.17 | 0.74 | -0.27 | 0.38 | 0.84 |
| Social contact (no mum and/or dad) | -0.31 | 0.17 | 0.07 | -0.64 | 0.02 | 0.19 |
| Year*Gender (female) | 0.00 | 0.02 | 0.92 | -0.05 | 0.04 | 0.95 |
| Year*Social contact (medium) | 0.01 | 0.02 | 0.66 | -0.03 | 0.05 | 0.82 |
| Year*Social contact (high) | 0.01 | 0.02 | 0.48 | -0.03 | 0.05 | 0.71 |
| Year*Social contact (no mum and/or dad) | 0.02 | 0.02 | 0.40 | -0.02 | 0.06 | 0.68 |
| Gender (female)*Social contact (medium) | -0.02 | 0.21 | 0.93 | -0.43 | 0.39 | 0.96 |
| Gender (female)*Social contact (high) | 0.10 | 0.22 | 0.64 | -0.33 | 0.54 | 0.81 |
| Gender (female)*Social contact (no mum and/or dad) | 0.19 | 0.21 | 0.38 | -0.23 | 0.61 | 0.68 |
| Year*Gender (female)*Social contact (medium) | 0.01 | 0.03 | 0.80 | -0.04 | 0.06 | 0.88 |
| Year*Gender (female)*Social contact (high) | 0.01 | 0.03 | 0.77 | -0.04 | 0.06 | 0.87 |
| Year*Gender (female)*Social contact (no mum and/or dad) | 0.03 | 0.03 | 0.20 | -0.02 | 0.08 | 0.45 |
| **Social contact with friends** |  |  |  |  |  |  |
| Intercept | 2.23 | 0.30 | 0.00 | 1.65 | 2.82 | 0.00 |
| Year | 0.38 | 0.01 | 0.00 | 0.35 | 0.41 | 0.00 |
| Gender (female) | 0.26 | 0.16 | 0.10 | -0.05 | 0.57 | 0.25 |
| Social contact (medium) | 0.13 | 0.13 | 0.28 | -0.11 | 0.38 | 0.59 |
| Social contact (high) | 0.03 | 0.13 | 0.81 | -0.23 | 0.29 | 0.88 |
| Year*Gender (female) | 0.00 | 0.02 | 0.85 | -0.04 | 0.04 | 0.91 |
| Year*Social contact (medium) | -0.04 | 0.02 | 0.01 | -0.07 | -0.01 | 0.06 |
| Year*Social contact (high) | -0.05 | 0.02 | 0.01 | -0.08 | -0.01 | 0.02 |
| Gender (female)*Social contact (medium) | -0.13 | 0.17 | 0.46 | -0.46 | 0.21 | 0.69 |
| Gender (female)*Social contact (high) | 0.02 | 0.17 | 0.93 | -0.33 | 0.36 | 0.96 |
| Year*Gender (female)*Social contact (medium) | 0.00 | 0.02 | 0.93 | -0.04 | 0.04 | 0.96 |
| Year*Gender (female)*Social contact (high) | 0.02 | 0.02 | 0.36 | -0.02 | 0.06 | 0.67 |
| **Relationship status** |  |  |  |  |  |  |
| Intercept | 2.46 | 0.30 | 0.00 | 1.87 | 3.04 | 0.00 |
| Year | 0.34 | 0.01 | 0.00 | 0.33 | 0.36 | 0.00 |
| Gender (female) | 0.20 | 0.08 | 0.01 | 0.04 | 0.36 | 0.06 |
| Relationship status (single) | -0.07 | 0.07 | 0.34 | -0.21 | 0.07 | 0.65 |
| Year*Gender (female) | 0.01 | 0.01 | 0.16 | -0.01 | 0.03 | 0.36 |
| Year*Relationship status (single) | 0.00 | 0.01 | 0.78 | -0.02 | 0.02 | 0.87 |
| Gender (female)*Relationship status (single) | -0.01 | 0.09 | 0.89 | -0.20 | 0.17 | 0.94 |
| Year*Gender (female)*Relationship status (single) | 0.00 | 0.01 | 0.89 | -0.02 | 0.02 | 0.94 |
| **Loneliness** |  |  |  |  |  |  |
| Intercept | 2.30 | 0.30 | 0.00 | 1.70 | 2.90 | 0.00 |
| Year | 0.34 | 0.01 | 0.00 | 0.33 | 0.35 | 0.00 |
| Gender (female) | 0.19 | 0.06 | 0.00 | 0.07 | 0.31 | 0.02 |
| Loneliness (medium) | -0.19 | 0.08 | 0.01 | -0.34 | -0.04 | 0.06 |
| Loneliness (high) | -0.06 | 0.15 | 0.70 | -0.34 | 0.23 | 0.82 |
| Year*Gender (female) | 0.01 | 0.01 | 0.12 | 0.00 | 0.03 | 0.28 |
| Year*Loneliness (medium) | 0.01 | 0.01 | 0.30 | -0.01 | 0.03 | 0.62 |
| Year*Loneliness (high) | 0.00 | 0.02 | 0.79 | -0.04 | 0.03 | 0.88 |
| Gender (female)*Loneliness (medium) | 0.08 | 0.11 | 0.46 | -0.13 | 0.29 | 0.69 |
| Gender (female)*Loneliness (high) | 0.03 | 0.18 | 0.89 | -0.33 | 0.38 | 0.94 |
| Year*Gender (female)*Loneliness (medium) | -0.01 | 0.01 | 0.70 | -0.03 | 0.02 | 0.82 |
| Year*Gender (female)*Loneliness (high) | 0.01 | 0.02 | 0.55 | -0.03 | 0.06 | 0.76 |
| **Perceived support from family** |  |  |  |  |  |  |
| Intercept | 2.45 | 0.40 | 0.00 | 1.67 | 3.23 | 0.00 |
| Year | 0.35 | 0.03 | 0.00 | 0.29 | 0.41 | 0.00 |
| Gender (female) | 0.12 | 0.30 | 0.69 | -0.47 | 0.71 | 0.82 |
| Perceived support (medium) | -0.06 | 0.24 | 0.81 | -0.52 | 0.41 | 0.88 |
| Perceived support (high) | 0.08 | 0.24 | 0.74 | -0.39 | 0.55 | 0.84 |
| Perceived support (no mum and/or dad) | -0.27 | 0.24 | 0.27 | -0.74 | 0.21 | 0.57 |
| Year*Gender (female) | 0.00 | 0.04 | 0.93 | -0.07 | 0.07 | 0.96 |
| Year*Perceived support (medium) | -0.01 | 0.03 | 0.87 | -0.07 | 0.06 | 0.92 |
| Year*Perceived support (high) | -0.01 | 0.03 | 0.84 | -0.07 | 0.06 | 0.91 |
| Year*Perceived support (no mum and/or dad) | 0.00 | 0.03 | 0.95 | -0.06 | 0.06 | 0.96 |
| Gender (female)*Perceived support (medium) | 0.04 | 0.31 | 0.89 | -0.57 | 0.66 | 0.94 |
| Gender (female)*Perceived support (high) | 0.04 | 0.31 | 0.90 | -0.57 | 0.64 | 0.94 |
| Gender (female)*Perceived support (no mum and/or dad) | 0.19 | 0.31 | 0.54 | -0.42 | 0.80 | 0.76 |
| Year*Gender (female)*Perceived support (medium) | 0.01 | 0.04 | 0.82 | -0.07 | 0.09 | 0.88 |
| Year*Gender (female)*Perceived support (high) | 0.00 | 0.04 | 0.94 | -0.08 | 0.07 | 0.96 |
| Year*Gender (female)*Perceived support (no mum and/or dad) | 0.03 | 0.04 | 0.47 | -0.05 | 0.10 | 0.69 |
| **Received support from family** |  |  |  |  |  |  |
| Intercept | 2.51 | 0.31 | 0.00 | 1.89 | 3.13 | 0.00 |
| Year | 0.34 | 0.01 | 0.00 | 0.32 | 0.35 | 0.00 |
| Gender (female) | 0.12 | 0.08 | 0.16 | -0.05 | 0.28 | 0.36 |
| Received support (medium) | 0.06 | 0.08 | 0.43 | -0.09 | 0.21 | 0.69 |
| Received support (high) | 0.17 | 0.19 | 0.36 | -0.20 | 0.55 | 0.67 |
| Received support (no mum and/or dad) | -0.29 | 0.09 | 0.00 | -0.46 | -0.12 | 0.01 |
| Year*Gender (female) | 0.01 | 0.01 | 0.55 | -0.01 | 0.03 | 0.76 |
| Year*Received support (medium) | 0.00 | 0.01 | 0.69 | -0.02 | 0.02 | 0.82 |
| Year*Received support (high) | 0.03 | 0.02 | 0.13 | -0.01 | 0.08 | 0.30 |
| Year*Received support (no mum and/or dad) | 0.01 | 0.01 | 0.31 | -0.01 | 0.03 | 0.64 |
| Gender (female)*Received support (medium) | 0.01 | 0.11 | 0.91 | -0.20 | 0.23 | 0.95 |
| Gender (female)*Received support (high) | 0.35 | 0.26 | 0.19 | -0.17 | 0.86 | 0.42 |
| Gender (female)*Received support (no mum and/or dad) | 0.20 | 0.11 | 0.08 | -0.02 | 0.41 | 0.20 |
| Year*Gender (female)*Received support (medium) | -0.01 | 0.01 | 0.71 | -0.03 | 0.02 | 0.82 |
| Year*Gender (female)*Received support (high) | -0.01 | 0.03 | 0.64 | -0.07 | 0.05 | 0.81 |
| Year*Gender (female)*Received support (no mum and/or dad) | 0.02 | 0.01 | 0.08 | 0.00 | 0.05 | 0.21 |
| **Perceived support from friends** |  |  |  |  |  |  |
| Intercept | 2.14 | 0.34 | 0.00 | 1.47 | 2.81 | 0.00 |
| Year | 0.37 | 0.03 | 0.00 | 0.31 | 0.42 | 0.00 |
| Gender (female) | 0.11 | 0.29 | 0.71 | -0.47 | 0.69 | 0.82 |
| Perceived support (medium) | 0.15 | 0.21 | 0.46 | -0.25 | 0.56 | 0.69 |
| Perceived support (high) | 0.23 | 0.21 | 0.27 | -0.18 | 0.65 | 0.57 |
| Year*Gender (female) | 0.04 | 0.04 | 0.34 | -0.04 | 0.11 | 0.65 |
| Year*Perceived support (medium) | -0.02 | 0.03 | 0.43 | -0.08 | 0.03 | 0.69 |
| Year*Perceived support (high) | -0.03 | 0.03 | 0.28 | -0.08 | 0.02 | 0.59 |
| Gender (female)*Perceived support (medium) | 0.02 | 0.30 | 0.94 | -0.57 | 0.61 | 0.96 |
| Gender (female)*Perceived support (high) | 0.13 | 0.30 | 0.67 | -0.46 | 0.72 | 0.82 |
| Year*Gender (female)*Perceived support (medium) | -0.01 | 0.04 | 0.73 | -0.09 | 0.07 | 0.84 |
| Year*Gender (female)*Perceived support (high) | -0.03 | 0.04 | 0.41 | -0.11 | 0.04 | 0.68 |
| **Received support from friends** |  |  |  |  |  |  |
| Intercept | 2.30 | 0.30 | 0.00 | 1.72 | 2.89 | 0.00 |
| Year | 0.35 | 0.01 | 0.00 | 0.33 | 0.36 | 0.00 |
| Gender (female) | 0.15 | 0.09 | 0.08 | -0.02 | 0.32 | 0.21 |
| Received support (medium) | 0.07 | 0.08 | 0.39 | -0.09 | 0.22 | 0.68 |
| Received support (high) | 0.11 | 0.09 | 0.21 | -0.06 | 0.28 | 0.46 |
| Year*Gender (female) | 0.03 | 0.01 | 0.00 | 0.01 | 0.05 | 0.01 |
| Year*Received support (medium) | -0.01 | 0.01 | 0.49 | -0.03 | 0.01 | 0.71 |
| Year*Received support (high) | -0.01 | 0.01 | 0.39 | -0.03 | 0.01 | 0.68 |
| Gender (female)*Received support (medium) | 0.06 | 0.11 | 0.58 | -0.16 | 0.29 | 0.77 |
| Gender (female)*Received support (high) | 0.00 | 0.12 | 0.98 | -0.23 | 0.24 | 0.99 |
| Year*Gender (female)*Received support (medium) | -0.02 | 0.01 | 0.08 | -0.05 | 0.00 | 0.22 |
| Year*Gender (female)*Received support (high) | -0.02 | 0.02 | 0.21 | -0.05 | 0.01 | 0.46 |
| **Positive experiences with family** |  |  |  |  |  |  |
| Intercept | 2.36 | 0.37 | 0.00 | 1.62 | 3.10 | 0.00 |
| Year | 0.33 | 0.03 | 0.00 | 0.28 | 0.38 | 0.00 |
| Gender (female) | -0.01 | 0.26 | 0.98 | -0.51 | 0.50 | 0.98 |
| Positive (medium) | 0.00 | 0.22 | 0.99 | -0.44 | 0.44 | 0.99 |
| Positive (high) | 0.12 | 0.21 | 0.58 | -0.30 | 0.53 | 0.77 |
| Positive (no mum and/or dad) | -0.24 | 0.21 | 0.27 | -0.65 | 0.18 | 0.57 |
| Year*Gender (female) | 0.01 | 0.03 | 0.78 | -0.05 | 0.07 | 0.87 |
| Year*Positive (medium) | 0.01 | 0.03 | 0.79 | -0.05 | 0.06 | 0.88 |
| Year*Positive (high) | 0.02 | 0.03 | 0.52 | -0.03 | 0.07 | 0.74 |
| Year*Positive (no mum and/or dad) | 0.02 | 0.03 | 0.41 | -0.03 | 0.07 | 0.68 |
| Gender (female)*Positive (medium) | 0.14 | 0.28 | 0.63 | -0.42 | 0.69 | 0.81 |
| Gender (female)*Positive (high) | 0.21 | 0.27 | 0.44 | -0.32 | 0.73 | 0.69 |
| Gender (female)*Positive (no mum and/or dad) | 0.32 | 0.26 | 0.22 | -0.19 | 0.83 | 0.48 |
| Year*Gender (female)*Positive (medium) | 0.00 | 0.03 | 0.99 | -0.07 | 0.07 | 0.99 |
| Year*Gender (female)*Positive (high) | -0.01 | 0.03 | 0.85 | -0.07 | 0.06 | 0.91 |
| Year*Gender (female)*Positive (no mum and/or dad) | 0.02 | 0.03 | 0.48 | -0.04 | 0.08 | 0.70 |
| **Negative experiences with family** |  |  |  |  |  |  |
| Intercept | 2.55 | 0.31 | 0.00 | 1.93 | 3.17 | 0.00 |
| Year | 0.34 | 0.01 | 0.00 | 0.32 | 0.35 | 0.00 |
| Gender (female) | 0.08 | 0.07 | 0.25 | -0.06 | 0.23 | 0.53 |
| Negative (medium) negative | -0.04 | 0.11 | 0.74 | -0.25 | 0.18 | 0.84 |
| Negative (high) negative | -0.05 | 0.11 | 0.68 | -0.27 | 0.17 | 0.82 |
| Negative (no mum and/or dad) | -0.33 | 0.09 | 0.00 | -0.50 | -0.16 | 0.00 |
| Year*Gender (female) | 0.01 | 0.01 | 0.36 | -0.01 | 0.03 | 0.67 |
| Year*Negative (medium) negative | 0.00 | 0.01 | 0.81 | -0.02 | 0.03 | 0.88 |
| Year*Negative (high) negative | 0.01 | 0.01 | 0.41 | -0.01 | 0.04 | 0.68 |
| Year*Negative (no mum and/or dad) | 0.01 | 0.01 | 0.34 | -0.01 | 0.03 | 0.65 |
| Gender (female)*Negative (medium) negative | 0.17 | 0.14 | 0.21 | -0.10 | 0.44 | 0.46 |
| Gender (female)*Negative (high) negative | 0.16 | 0.15 | 0.30 | -0.14 | 0.46 | 0.62 |
| Gender (female)*Negative (no mum and/or dad) | 0.23 | 0.10 | 0.03 | 0.02 | 0.43 | 0.10 |
| Year*Gender (female)*Negative (medium) negative | -0.01 | 0.02 | 0.41 | -0.05 | 0.02 | 0.68 |
| Year*Gender (female)*Negative (high) negative | -0.01 | 0.02 | 0.51 | -0.05 | 0.02 | 0.73 |
| Year*Gender (female)*Negative (no mum and/or dad) | 0.02 | 0.01 | 0.10 | 0.00 | 0.05 | 0.25 |

Note. This table represents findings from linear mixed effect models controlling for age, ethnicity, baseline BMI, neighbourhood disadvantage, parental education and occupation, and self-rated health. *FDR stands for false discovery rate adjustment, using the Benjamini Hochberg procedure

## Supplementary index 9: Results for growth curve model by ethnicity.

| **Term** | **Estimate** | **Std error** | **P value** | **2.5 %** | **97.5 %** | **P value FDR** |
| --- | --- | --- | --- | --- | --- | --- |
| **Social contact with family** |  |  |  |  |  |  |
| Intercept | 2.32 | 0.33 | 0.00 | 1.67 | 2.97 | 0.00 |
| Year | 0.32 | 0.02 | 0.00 | 0.29 | 0.35 | 0.00 |
| Ethnicity (Hispanic) | -0.16 | 0.29 | 0.58 | -0.74 | 0.41 | 0.87 |
| Ethnicity (Non-Hispanic Asian) | -0.01 | 0.32 | 0.98 | -0.64 | 0.63 | 0.99 |
| Ethnicity (Non-Hispanic Black) | 0.09 | 0.28 | 0.76 | -0.47 | 0.64 | 0.91 |
| Social contact (medium) | 0.03 | 0.13 | 0.84 | -0.24 | 0.29 | 0.95 |
| Social contact (high) | 0.14 | 0.14 | 0.33 | -0.14 | 0.42 | 0.76 |
| Social contact (no mum and/or dad) | -0.14 | 0.15 | 0.36 | -0.43 | 0.16 | 0.78 |
| Year*Ethnicity (Hispanic) | 0.04 | 0.04 | 0.31 | -0.03 | 0.11 | 0.74 |
| Year*Ethnicity (Non-Hispanic Asian) | -0.03 | 0.04 | 0.49 | -0.11 | 0.05 | 0.85 |
| Year*Ethnicity (Non-Hispanic Black) | 0.05 | 0.03 | 0.12 | -0.01 | 0.12 | 0.34 |
| Year*Social contact (medium) | 0.01 | 0.02 | 0.46 | -0.02 | 0.04 | 0.83 |
| Year*Social contact (high) | 0.02 | 0.02 | 0.35 | -0.02 | 0.05 | 0.78 |
| Year*Social contact (no mum and/or dad) | 0.03 | 0.02 | 0.15 | -0.01 | 0.06 | 0.41 |
| Ethnicity (Hispanic)*Social contact (medium) | -0.02 | 0.31 | 0.96 | -0.63 | 0.60 | 0.98 |
| Ethnicity (Non-Hispanic Asian)*Social contact (medium) | -0.13 | 0.36 | 0.72 | -0.85 | 0.58 | 0.88 |
| Ethnicity (Non-Hispanic Black)*Social contact (medium) | -0.20 | 0.29 | 0.50 | -0.76 | 0.37 | 0.85 |
| Ethnicity (Hispanic)*Social contact (high) | 0.15 | 0.31 | 0.64 | -0.47 | 0.77 | 0.87 |
| Ethnicity (Non-Hispanic Asian)*Social contact (high) | -0.37 | 0.37 | 0.33 | -1.10 | 0.37 | 0.76 |
| Ethnicity (Non-Hispanic Black)*Social contact (high) | -0.08 | 0.31 | 0.79 | -0.69 | 0.53 | 0.92 |
| Ethnicity (Hispanic)*Social contact (no mum and/or dad) | 0.14 | 0.31 | 0.65 | -0.47 | 0.74 | 0.87 |
| Ethnicity (Non-Hispanic Asian)*Social contact (no mum and/or dad) | -0.10 | 0.39 | 0.80 | -0.86 | 0.67 | 0.92 |
| Ethnicity (Non-Hispanic Black)*Social contact (no mum and/or dad) | -0.16 | 0.29 | 0.59 | -0.74 | 0.42 | 0.87 |
| Year*Ethnicity (Hispanic)*Social contact (medium) | 0.00 | 0.04 | 0.99 | -0.08 | 0.08 | 0.99 |
| Year*Ethnicity (Non-Hispanic Asian)*Social contact (medium) | 0.00 | 0.04 | 0.94 | -0.08 | 0.09 | 0.97 |
| Year*Ethnicity (Non-Hispanic Black)*Social contact (medium) | 0.00 | 0.03 | 0.99 | -0.07 | 0.07 | 0.99 |
| Year*Ethnicity (Hispanic)*Social contact (high) | 0.00 | 0.04 | 0.97 | -0.08 | 0.07 | 0.98 |
| Year*Ethnicity (Non-Hispanic Asian)*Social contact (high) | 0.00 | 0.05 | 0.92 | -0.09 | 0.10 | 0.96 |
| Year*Ethnicity (Non-Hispanic Black)*Social contact (high) | 0.01 | 0.04 | 0.89 | -0.07 | 0.08 | 0.96 |
| Year*Ethnicity (Hispanic)*Social contact (no mum and/or dad) | -0.01 | 0.04 | 0.86 | -0.08 | 0.07 | 0.95 |
| Year*Ethnicity (Non-Hispanic Asian)*Social contact (no mum and/or dad) | -0.02 | 0.05 | 0.65 | -0.12 | 0.07 | 0.87 |
| Year*Ethnicity (Non-Hispanic Black)*Social contact (no mum and/or dad) | -0.01 | 0.04 | 0.88 | -0.08 | 0.07 | 0.95 |
| **Social contact with friends** |  |  |  |  |  |  |
| Intercept | 2.20 | 0.29 | 0.00 | 1.63 | 2.78 | 0.00 |
| Year | 0.36 | 0.01 | 0.00 | 0.33 | 0.39 | 0.00 |
| Ethnicity (Hispanic) | -0.14 | 0.22 | 0.52 | -0.58 | 0.29 | 0.87 |
| Ethnicity (Non-Hispanic Asian) | -0.34 | 0.34 | 0.32 | -1.00 | 0.33 | 0.75 |
| Ethnicity (Non-Hispanic Black) | 0.28 | 0.19 | 0.16 | -0.11 | 0.66 | 0.43 |
| Social contact (medium) | 0.13 | 0.12 | 0.27 | -0.10 | 0.37 | 0.67 |
| Social contact (high) | 0.09 | 0.12 | 0.43 | -0.14 | 0.33 | 0.82 |
| Year*Ethnicity (Hispanic) | 0.04 | 0.03 | 0.16 | -0.02 | 0.09 | 0.44 |
| Year*Ethnicity (Non-Hispanic Asian) | -0.04 | 0.04 | 0.37 | -0.12 | 0.04 | 0.78 |
| Year*Ethnicity (Non-Hispanic Black) | 0.07 | 0.02 | 0.01 | 0.02 | 0.11 | 0.05 |
| Year*Social contact (medium) | -0.03 | 0.01 | 0.07 | -0.06 | 0.00 | 0.22 |
| Year*Social contact (high) | -0.03 | 0.02 | 0.04 | -0.06 | 0.00 | 0.16 |
| Ethnicity (Hispanic)*Social contact (medium) | 0.12 | 0.24 | 0.63 | -0.37 | 0.60 | 0.87 |
| Ethnicity (Non-Hispanic Asian)*Social contact (medium) | 0.05 | 0.36 | 0.90 | -0.67 | 0.76 | 0.96 |
| Ethnicity (Non-Hispanic Black)*Social contact (medium) | -0.43 | 0.21 | 0.04 | -0.84 | -0.01 | 0.17 |
| Ethnicity (Hispanic)*Social contact (high) | 0.03 | 0.26 | 0.92 | -0.49 | 0.54 | 0.96 |
| Ethnicity (Non-Hispanic Asian)*Social contact (high) | 0.37 | 0.38 | 0.33 | -0.37 | 1.11 | 0.76 |
| Ethnicity (Non-Hispanic Black)*Social contact (high) | -0.42 | 0.21 | 0.04 | -0.83 | -0.01 | 0.17 |
| Year*Ethnicity (Hispanic)*Social contact (medium) | -0.01 | 0.03 | 0.61 | -0.07 | 0.04 | 0.87 |
| Year*Ethnicity (Non-Hispanic Asian)*Social contact (medium) | 0.00 | 0.04 | 0.95 | -0.09 | 0.09 | 0.97 |
| Year*Ethnicity (Non-Hispanic Black)*Social contact (medium) | -0.01 | 0.03 | 0.61 | -0.07 | 0.04 | 0.87 |
| Year*Ethnicity (Hispanic)*Social contact (high) | 0.01 | 0.03 | 0.76 | -0.05 | 0.07 | 0.91 |
| Year*Ethnicity (Non-Hispanic Asian)*Social contact (high) | 0.01 | 0.04 | 0.76 | -0.07 | 0.10 | 0.91 |
| Year*Ethnicity (Non-Hispanic Black)*Social contact (high) | -0.01 | 0.03 | 0.71 | -0.06 | 0.04 | 0.88 |
| **Relationship status** |  |  |  |  |  |  |
| Intercept | 2.40 | 0.31 | 0.00 | 1.79 | 3.02 | 0.00 |
| Year | 0.33 | 0.01 | 0.00 | 0.32 | 0.35 | 0.00 |
| Ethnicity (Hispanic) | 0.06 | 0.12 | 0.62 | -0.17 | 0.29 | 0.87 |
| Ethnicity (Non-Hispanic Asian) | -0.03 | 0.22 | 0.91 | -0.45 | 0.40 | 0.96 |
| Ethnicity (Non-Hispanic Black) | -0.18 | 0.10 | 0.07 | -0.37 | 0.01 | 0.23 |
| Relationship status (single) | -0.06 | 0.07 | 0.36 | -0.19 | 0.07 | 0.78 |
| Year*Ethnicity (Hispanic) | 0.04 | 0.01 | 0.01 | 0.01 | 0.06 | 0.05 |
| Year*Ethnicity (Non-Hispanic Asian) | 0.00 | 0.03 | 0.85 | -0.04 | 0.05 | 0.95 |
| Year*Ethnicity (Non-Hispanic Black) | 0.05 | 0.01 | 0.00 | 0.03 | 0.07 | 0.00 |
| Year*Relationship status (single) | 0.00 | 0.01 | 0.99 | -0.02 | 0.02 | 0.99 |
| Ethnicity (Hispanic)*Relationship status (single) | -0.21 | 0.14 | 0.14 | -0.49 | 0.07 | 0.40 |
| Ethnicity (Non-Hispanic Asian)*Relationship status (single) | -0.18 | 0.25 | 0.46 | -0.67 | 0.30 | 0.83 |
| Ethnicity (Non-Hispanic Black)*Relationship status (single) | 0.11 | 0.12 | 0.35 | -0.12 | 0.34 | 0.78 |
| Year*Ethnicity (Hispanic)*Relationship status (single) | 0.00 | 0.02 | 0.78 | -0.04 | 0.03 | 0.92 |
| Year*Ethnicity (Non-Hispanic Asian)*Relationship status (single) | -0.05 | 0.03 | 0.11 | -0.10 | 0.01 | 0.32 |
| Year*Ethnicity (Non-Hispanic Black)*Relationship status (single) | 0.01 | 0.01 | 0.62 | -0.02 | 0.03 | 0.87 |
| **Loneliness** |  |  |  |  |  |  |
| Intercept | 2.28 | 0.29 | 0.00 | 1.70 | 2.86 | 0.00 |
| Year | 0.33 | 0.01 | 0.00 | 0.32 | 0.34 | 0.00 |
| Ethnicity (Hispanic) | -0.19 | 0.09 | 0.05 | -0.37 | 0.00 | 0.18 |
| Ethnicity (Non-Hispanic Asian) | -0.23 | 0.13 | 0.09 | -0.49 | 0.04 | 0.29 |
| Ethnicity (Non-Hispanic Black) | -0.10 | 0.08 | 0.24 | -0.27 | 0.07 | 0.61 |
| Loneliness (medium) | -0.22 | 0.07 | 0.00 | -0.35 | -0.08 | 0.02 |
| Loneliness (high) | -0.07 | 0.12 | 0.58 | -0.31 | 0.17 | 0.87 |
| Year*Ethnicity (Hispanic) | 0.03 | 0.01 | 0.00 | 0.01 | 0.05 | 0.03 |
| Year*Ethnicity (Non-Hispanic Asian) | -0.04 | 0.02 | 0.03 | -0.07 | 0.00 | 0.13 |
| Year*Ethnicity (Non-Hispanic Black) | 0.05 | 0.01 | 0.00 | 0.03 | 0.07 | 0.00 |
| Year*Loneliness (medium) | 0.00 | 0.01 | 0.80 | -0.02 | 0.02 | 0.92 |
| Year*Loneliness (high) | 0.01 | 0.02 | 0.69 | -0.02 | 0.04 | 0.87 |
| Ethnicity (Hispanic)*Loneliness (medium) | 0.35 | 0.15 | 0.02 | 0.05 | 0.65 | 0.13 |
| Ethnicity (Non-Hispanic Asian)*Loneliness (medium) | 0.12 | 0.22 | 0.59 | -0.31 | 0.54 | 0.87 |
| Ethnicity (Non-Hispanic Black)*Loneliness (medium) | 0.00 | 0.14 | 0.99 | -0.27 | 0.28 | 0.99 |
| Ethnicity (Hispanic)*Loneliness (high) | 0.13 | 0.25 | 0.61 | -0.37 | 0.63 | 0.87 |
| Ethnicity (Non-Hispanic Asian)*Loneliness (high) | 0.36 | 0.39 | 0.36 | -0.41 | 1.14 | 0.78 |
| Ethnicity (Non-Hispanic Black)*Loneliness (high) | -0.03 | 0.21 | 0.89 | -0.45 | 0.39 | 0.96 |
| Year*Ethnicity (Hispanic)*Loneliness (medium) | 0.01 | 0.02 | 0.77 | -0.03 | 0.04 | 0.91 |
| Year*Ethnicity (Non-Hispanic Asian)*Loneliness (medium) | 0.02 | 0.03 | 0.56 | -0.04 | 0.07 | 0.87 |
| Year*Ethnicity (Non-Hispanic Black)*Loneliness (medium) | 0.02 | 0.02 | 0.24 | -0.01 | 0.05 | 0.61 |
| Year*Ethnicity (Hispanic)*Loneliness (high) | 0.00 | 0.03 | 0.93 | -0.05 | 0.06 | 0.96 |
| Year*Ethnicity (Non-Hispanic Asian)*Loneliness (high) | 0.01 | 0.05 | 0.76 | -0.08 | 0.11 | 0.91 |
| Year*Ethnicity (Non-Hispanic Black)*Loneliness (high) | -0.02 | 0.03 | 0.41 | -0.07 | 0.03 | 0.80 |
| **Perceived support from family** |  |  |  |  |  |  |
| Intercept | 2.32 | 0.36 | 0.00 | 1.61 | 3.02 | 0.00 |
| Year | 0.34 | 0.03 | 0.00 | 0.29 | 0.39 | 0.00 |
| Ethnicity (Hispanic) | 0.24 | 0.46 | 0.60 | -0.66 | 1.14 | 0.87 |
| Ethnicity (Non-Hispanic Asian) | 0.18 | 0.53 | 0.73 | -0.86 | 1.22 | 0.89 |
| Ethnicity (Non-Hispanic Black) | -0.35 | 0.46 | 0.45 | -1.25 | 0.56 | 0.83 |
| Perceived support (medium) | -0.02 | 0.21 | 0.92 | -0.42 | 0.38 | 0.96 |
| Perceived support (high) | 0.16 | 0.20 | 0.44 | -0.24 | 0.56 | 0.83 |
| Perceived support (no mum and/or dad) | -0.10 | 0.21 | 0.64 | -0.50 | 0.31 | 0.87 |
| Year*Ethnicity (Hispanic) | 0.03 | 0.05 | 0.65 | -0.08 | 0.13 | 0.87 |
| Year*Ethnicity (Non-Hispanic Asian) | 0.00 | 0.06 | 0.98 | -0.13 | 0.12 | 0.99 |
| Year*Ethnicity (Non-Hispanic Black) | 0.03 | 0.05 | 0.60 | -0.08 | 0.14 | 0.87 |
| Year*Perceived support (medium) | 0.00 | 0.03 | 0.87 | -0.06 | 0.05 | 0.95 |
| Year*Perceived support (high) | -0.01 | 0.03 | 0.68 | -0.06 | 0.04 | 0.87 |
| Year*Perceived support (no mum and/or dad) | 0.01 | 0.03 | 0.85 | -0.05 | 0.06 | 0.95 |
| Ethnicity (Hispanic)*Perceived support (medium) | -0.20 | 0.49 | 0.67 | -1.16 | 0.75 | 0.87 |
| Ethnicity (Non-Hispanic Asian)*Perceived support (medium) | -0.25 | 0.56 | 0.66 | -1.36 | 0.86 | 0.87 |
| Ethnicity (Non-Hispanic Black)*Perceived support (medium) | 0.26 | 0.48 | 0.58 | -0.68 | 1.21 | 0.87 |
| Ethnicity (Hispanic)*Perceived support (high) | -0.42 | 0.47 | 0.38 | -1.35 | 0.52 | 0.78 |
| Ethnicity (Non-Hispanic Asian)*Perceived support (high) | -0.49 | 0.55 | 0.38 | -1.58 | 0.60 | 0.78 |
| Ethnicity (Non-Hispanic Black)*Perceived support (high) | 0.32 | 0.47 | 0.50 | -0.61 | 1.25 | 0.85 |
| Ethnicity (Hispanic)*Perceived support (no mum and/or dad) | -0.26 | 0.47 | 0.58 | -1.18 | 0.66 | 0.87 |
| Ethnicity (Non-Hispanic Asian)*Perceived support (no mum and/or dad) | -0.26 | 0.59 | 0.66 | -1.41 | 0.89 | 0.87 |
| Ethnicity (Non-Hispanic Black)*Perceived support (no mum and/or dad) | 0.26 | 0.47 | 0.58 | -0.66 | 1.19 | 0.87 |
| Year*Ethnicity (Hispanic)*Perceived support (medium) | 0.02 | 0.06 | 0.73 | -0.10 | 0.14 | 0.89 |
| Year*Ethnicity (Non-Hispanic Asian)*Perceived support (medium) | -0.04 | 0.07 | 0.61 | -0.17 | 0.10 | 0.87 |
| Year*Ethnicity (Non-Hispanic Black)*Perceived support (medium) | 0.03 | 0.06 | 0.64 | -0.09 | 0.14 | 0.87 |
| Year*Ethnicity (Hispanic)*Perceived support (high) | 0.01 | 0.06 | 0.92 | -0.11 | 0.12 | 0.96 |
| Year*Ethnicity (Non-Hispanic Asian)*Perceived support (high) | -0.02 | 0.07 | 0.77 | -0.15 | 0.11 | 0.91 |
| Year*Ethnicity (Non-Hispanic Black)*Perceived support (high) | 0.03 | 0.06 | 0.62 | -0.08 | 0.14 | 0.87 |
| Year*Ethnicity (Hispanic)*Perceived support (no mum and/or dad) | 0.00 | 0.06 | 0.93 | -0.11 | 0.12 | 0.97 |
| Year*Ethnicity (Non-Hispanic Asian)*Perceived support (no mum and/or dad) | -0.05 | 0.07 | 0.51 | -0.18 | 0.09 | 0.86 |
| Year*Ethnicity (Non-Hispanic Black)*Perceived support (no mum and/or dad) | 0.02 | 0.06 | 0.73 | -0.09 | 0.13 | 0.89 |
| **Received support from family** |  |  |  |  |  |  |
| Intercept | 2.41 | 0.30 | 0.00 | 1.81 | 3.01 | 0.00 |
| Year | 0.33 | 0.01 | 0.00 | 0.32 | 0.34 | 0.00 |
| Ethnicity (Hispanic) | -0.06 | 0.12 | 0.61 | -0.29 | 0.17 | 0.87 |
| Ethnicity (Non-Hispanic Asian) | -0.25 | 0.15 | 0.09 | -0.53 | 0.04 | 0.30 |
| Ethnicity (Non-Hispanic Black) | -0.11 | 0.13 | 0.40 | -0.36 | 0.14 | 0.79 |
| Received support (medium) | 0.05 | 0.07 | 0.49 | -0.09 | 0.18 | 0.85 |
| Received support (high) | 0.28 | 0.15 | 0.06 | -0.01 | 0.57 | 0.20 |
| Received support (no mum and/or dad) | -0.17 | 0.08 | 0.04 | -0.33 | 0.00 | 0.18 |
| Year*Ethnicity (Hispanic) | 0.03 | 0.01 | 0.03 | 0.00 | 0.06 | 0.14 |
| Year*Ethnicity (Non-Hispanic Asian) | -0.03 | 0.02 | 0.05 | -0.07 | 0.00 | 0.18 |
| Year*Ethnicity (Non-Hispanic Black) | 0.04 | 0.01 | 0.00 | 0.02 | 0.07 | 0.02 |
| Year*Received support (medium) | 0.00 | 0.01 | 0.65 | -0.02 | 0.01 | 0.87 |
| Year*Received support (high) | 0.02 | 0.02 | 0.38 | -0.02 | 0.05 | 0.78 |
| Year*Received support (no mum and/or dad) | 0.01 | 0.01 | 0.19 | -0.01 | 0.03 | 0.50 |
| Ethnicity (Hispanic)*Received support (medium) | -0.08 | 0.16 | 0.62 | -0.40 | 0.24 | 0.87 |
| Ethnicity (Non-Hispanic Asian)*Received support (medium) | 0.13 | 0.24 | 0.60 | -0.34 | 0.59 | 0.87 |
| Ethnicity (Non-Hispanic Black)*Received support (medium) | 0.05 | 0.17 | 0.77 | -0.28 | 0.38 | 0.91 |
| Ethnicity (Hispanic)*Received support (high) | -0.02 | 0.39 | 0.96 | -0.78 | 0.74 | 0.98 |
| Ethnicity (Non-Hispanic Asian)*Received support (high) | -0.17 | 0.58 | 0.77 | -1.32 | 0.98 | 0.91 |
| Ethnicity (Non-Hispanic Black)*Received support (high) | 0.53 | 0.37 | 0.15 | -0.19 | 1.25 | 0.42 |
| Ethnicity (Hispanic)*Received support (no mum and/or dad) | 0.03 | 0.16 | 0.84 | -0.28 | 0.34 | 0.95 |
| Ethnicity (Non-Hispanic Asian)*Received support (no mum and/or dad) | 0.14 | 0.28 | 0.60 | -0.40 | 0.69 | 0.87 |
| Ethnicity (Non-Hispanic Black)*Received support (no mum and/or dad) | 0.03 | 0.15 | 0.85 | -0.27 | 0.33 | 0.95 |
| Year*Ethnicity (Hispanic)*Received support (medium) | 0.01 | 0.02 | 0.67 | -0.03 | 0.05 | 0.87 |
| Year*Ethnicity (Non-Hispanic Asian)*Received support (medium) | 0.02 | 0.03 | 0.39 | -0.03 | 0.08 | 0.79 |
| Year*Ethnicity (Non-Hispanic Black)*Received support (medium) | 0.02 | 0.02 | 0.45 | -0.02 | 0.06 | 0.83 |
| Year*Ethnicity (Hispanic)*Received support (high) | 0.01 | 0.05 | 0.86 | -0.08 | 0.10 | 0.95 |
| Year*Ethnicity (Non-Hispanic Asian)*Received support (high) | -0.01 | 0.07 | 0.90 | -0.15 | 0.13 | 0.96 |
| Year*Ethnicity (Non-Hispanic Black)*Received support (high) | 0.06 | 0.04 | 0.17 | -0.02 | 0.14 | 0.45 |
| Year*Ethnicity (Hispanic)*Received support (no mum and/or dad) | 0.00 | 0.02 | 0.91 | -0.04 | 0.03 | 0.96 |
| Year*Ethnicity (Non-Hispanic Asian)*Received support (no mum and/or dad) | -0.02 | 0.03 | 0.59 | -0.08 | 0.04 | 0.87 |
| Year*Ethnicity (Non-Hispanic Black)*Received support (no mum and/or dad) | 0.00 | 0.02 | 0.84 | -0.03 | 0.04 | 0.95 |
| **Perceived support from friends** |  |  |  |  |  |  |
| Intercept | 2.03 | 0.38 | 0.00 | 1.28 | 2.78 | 0.00 |
| Year | 0.35 | 0.03 | 0.00 | 0.29 | 0.42 | 0.00 |
| Ethnicity (Hispanic) | 0.36 | 0.40 | 0.36 | -0.42 | 1.15 | 0.78 |
| Ethnicity (Non-Hispanic Asian) | -0.52 | 0.60 | 0.38 | -1.69 | 0.65 | 0.78 |
| Ethnicity (Non-Hispanic Black) | -0.06 | 0.33 | 0.86 | -0.71 | 0.60 | 0.95 |
| Perceived support (medium) | 0.20 | 0.26 | 0.44 | -0.30 | 0.70 | 0.83 |
| Perceived support (high) | 0.37 | 0.26 | 0.16 | -0.14 | 0.89 | 0.43 |
| Year*Ethnicity (Hispanic) | 0.06 | 0.05 | 0.22 | -0.04 | 0.16 | 0.57 |
| Year*Ethnicity (Non-Hispanic Asian) | 0.02 | 0.08 | 0.83 | -0.14 | 0.17 | 0.94 |
| Year*Ethnicity (Non-Hispanic Black) | 0.03 | 0.04 | 0.45 | -0.05 | 0.12 | 0.83 |
| Year*Perceived support (medium) | -0.02 | 0.03 | 0.62 | -0.08 | 0.05 | 0.87 |
| Year*Perceived support (high) | -0.03 | 0.03 | 0.38 | -0.09 | 0.04 | 0.78 |
| Ethnicity (Hispanic)*Perceived support (medium) | -0.35 | 0.41 | 0.40 | -1.16 | 0.46 | 0.79 |
| Ethnicity (Non-Hispanic Asian)*Perceived support (medium) | 0.45 | 0.62 | 0.47 | -0.77 | 1.66 | 0.83 |
| Ethnicity (Non-Hispanic Black)*Perceived support (medium) | -0.02 | 0.35 | 0.95 | -0.71 | 0.67 | 0.98 |
| Ethnicity (Hispanic)*Perceived support (high) | -0.58 | 0.43 | 0.18 | -1.43 | 0.27 | 0.49 |
| Ethnicity (Non-Hispanic Asian)*Perceived support (high) | 0.27 | 0.62 | 0.66 | -0.95 | 1.49 | 0.87 |
| Ethnicity (Non-Hispanic Black)*Perceived support (high) | -0.05 | 0.34 | 0.89 | -0.73 | 0.63 | 0.96 |
| Year*Ethnicity (Hispanic)*Perceived support (medium) | -0.03 | 0.05 | 0.61 | -0.12 | 0.07 | 0.87 |
| Year*Ethnicity (Non-Hispanic Asian)*Perceived support (medium) | -0.05 | 0.08 | 0.55 | -0.21 | 0.11 | 0.87 |
| Year*Ethnicity (Non-Hispanic Black)*Perceived support (medium) | 0.02 | 0.05 | 0.68 | -0.07 | 0.11 | 0.87 |
| Year*Ethnicity (Hispanic)*Perceived support (high) | -0.03 | 0.05 | 0.57 | -0.13 | 0.07 | 0.87 |
| Year*Ethnicity (Non-Hispanic Asian)*Perceived support (high) | -0.05 | 0.08 | 0.54 | -0.21 | 0.11 | 0.87 |
| Year*Ethnicity (Non-Hispanic Black)*Perceived support (high) | 0.03 | 0.04 | 0.53 | -0.06 | 0.12 | 0.87 |
| **Received support from friends** |  |  |  |  |  |  |
| Intercept | 2.27 | 0.28 | 0.00 | 1.71 | 2.83 | 0.00 |
| Year | 0.34 | 0.01 | 0.00 | 0.33 | 0.36 | 0.00 |
| Ethnicity (Hispanic) | 0.03 | 0.12 | 0.80 | -0.20 | 0.26 | 0.92 |
| Ethnicity (Non-Hispanic Asian) | -0.07 | 0.18 | 0.69 | -0.42 | 0.28 | 0.87 |
| Ethnicity (Non-Hispanic Black) | -0.17 | 0.11 | 0.11 | -0.38 | 0.04 | 0.32 |
| Received support (medium) | 0.05 | 0.08 | 0.51 | -0.10 | 0.20 | 0.86 |
| Received support (high) | 0.10 | 0.08 | 0.19 | -0.05 | 0.26 | 0.51 |
| Year*Ethnicity (Hispanic) | 0.02 | 0.02 | 0.16 | -0.01 | 0.05 | 0.45 |
| Year*Ethnicity (Non-Hispanic Asian) | -0.04 | 0.02 | 0.08 | -0.08 | 0.00 | 0.25 |
| Year*Ethnicity (Non-Hispanic Black) | 0.05 | 0.01 | 0.00 | 0.02 | 0.07 | 0.00 |
| Year*Received support (medium) | -0.02 | 0.01 | 0.12 | -0.03 | 0.00 | 0.33 |
| Year*Received support (high) | -0.02 | 0.01 | 0.08 | -0.04 | 0.00 | 0.26 |
| Ethnicity (Hispanic)*Received support (medium) | -0.05 | 0.16 | 0.76 | -0.37 | 0.27 | 0.91 |
| Ethnicity (Non-Hispanic Asian)*Received support (medium) | -0.33 | 0.25 | 0.20 | -0.83 | 0.17 | 0.52 |
| Ethnicity (Non-Hispanic Black)*Received support (medium) | 0.24 | 0.14 | 0.10 | -0.05 | 0.52 | 0.31 |
| Ethnicity (Hispanic)*Received support (high) | -0.27 | 0.17 | 0.11 | -0.59 | 0.06 | 0.32 |
| Ethnicity (Non-Hispanic Asian)*Received support (high) | 0.06 | 0.25 | 0.81 | -0.43 | 0.56 | 0.92 |
| Ethnicity (Non-Hispanic Black)*Received support (high) | -0.02 | 0.14 | 0.89 | -0.29 | 0.25 | 0.96 |
| Year*Ethnicity (Hispanic)*Received support (medium) | 0.01 | 0.02 | 0.60 | -0.03 | 0.05 | 0.87 |
| Year*Ethnicity (Non-Hispanic Asian)*Received support (medium) | 0.02 | 0.03 | 0.50 | -0.04 | 0.08 | 0.85 |
| Year*Ethnicity (Non-Hispanic Black)*Received support (medium) | 0.01 | 0.02 | 0.68 | -0.03 | 0.04 | 0.87 |
| Year*Ethnicity (Hispanic)*Received support (high) | 0.03 | 0.02 | 0.18 | -0.01 | 0.07 | 0.49 |
| Year*Ethnicity (Non-Hispanic Asian)*Received support (high) | 0.00 | 0.03 | 0.96 | -0.06 | 0.06 | 0.98 |
| Year*Ethnicity (Non-Hispanic Black)*Received support (high) | 0.02 | 0.02 | 0.25 | -0.01 | 0.05 | 0.62 |
| **Positive experiences with family** |  |  |  |  |  |  |
| Intercept | 2.13 | 0.34 | 0.00 | 1.45 | 2.80 | 0.00 |
| Year | 0.33 | 0.02 | 0.00 | 0.30 | 0.37 | 0.00 |
| Ethnicity (Hispanic) | -0.05 | 0.32 | 0.88 | -0.68 | 0.58 | 0.95 |
| Ethnicity (Non-Hispanic Asian) | 0.20 | 0.41 | 0.63 | -0.61 | 1.01 | 0.87 |
| Ethnicity (Non-Hispanic Black) | -0.23 | 0.38 | 0.55 | -0.98 | 0.52 | 0.87 |
| Positive (medium) | 0.11 | 0.17 | 0.53 | -0.22 | 0.43 | 0.87 |
| Positive (high) | 0.29 | 0.16 | 0.06 | -0.02 | 0.60 | 0.22 |
| Positive (no mum and/or dad) | 0.02 | 0.16 | 0.90 | -0.30 | 0.34 | 0.96 |
| Year*Ethnicity (Hispanic) | 0.01 | 0.04 | 0.79 | -0.06 | 0.08 | 0.92 |
| Year*Ethnicity (Non-Hispanic Asian) | -0.04 | 0.05 | 0.40 | -0.14 | 0.05 | 0.79 |
| Year*Ethnicity (Non-Hispanic Black) | -0.01 | 0.05 | 0.91 | -0.10 | 0.09 | 0.96 |
| Year*Positive (medium) | -0.01 | 0.02 | 0.59 | -0.05 | 0.03 | 0.87 |
| Year*Positive (high) | 0.00 | 0.02 | 0.90 | -0.04 | 0.04 | 0.96 |
| Year*Positive (no mum and/or dad) | 0.01 | 0.02 | 0.65 | -0.03 | 0.05 | 0.87 |
| Ethnicity (Hispanic)*Positive (medium) | 0.06 | 0.36 | 0.88 | -0.65 | 0.76 | 0.95 |
| Ethnicity (Non-Hispanic Asian)*Positive (medium) | -0.26 | 0.47 | 0.58 | -1.18 | 0.66 | 0.87 |
| Ethnicity (Non-Hispanic Black)*Positive (medium) | 0.04 | 0.43 | 0.92 | -0.80 | 0.88 | 0.96 |
| Ethnicity (Hispanic)*Positive (high) | -0.09 | 0.33 | 0.78 | -0.74 | 0.56 | 0.92 |
| Ethnicity (Non-Hispanic Asian)*Positive (high) | -0.52 | 0.44 | 0.24 | -1.39 | 0.35 | 0.61 |
| Ethnicity (Non-Hispanic Black)*Positive (high) | 0.23 | 0.40 | 0.57 | -0.56 | 1.02 | 0.87 |
| Ethnicity (Hispanic)*Positive (no mum and/or dad) | 0.02 | 0.33 | 0.95 | -0.63 | 0.67 | 0.97 |
| Ethnicity (Non-Hispanic Asian)*Positive (no mum and/or dad) | -0.31 | 0.47 | 0.52 | -1.23 | 0.62 | 0.87 |
| Ethnicity (Non-Hispanic Black)*Positive (no mum and/or dad) | 0.15 | 0.39 | 0.70 | -0.61 | 0.91 | 0.87 |
| Year*Ethnicity (Hispanic)*Positive (medium) | 0.04 | 0.04 | 0.28 | -0.04 | 0.12 | 0.67 |
| Year*Ethnicity (Non-Hispanic Asian)*Positive (medium) | 0.01 | 0.06 | 0.81 | -0.10 | 0.12 | 0.92 |
| Year*Ethnicity (Non-Hispanic Black)*Positive (medium) | 0.07 | 0.05 | 0.20 | -0.03 | 0.17 | 0.51 |
| Year*Ethnicity (Hispanic)*Positive (high) | 0.02 | 0.04 | 0.60 | -0.06 | 0.10 | 0.87 |
| Year*Ethnicity (Non-Hispanic Asian)*Positive (high) | 0.02 | 0.05 | 0.69 | -0.08 | 0.12 | 0.87 |
| Year*Ethnicity (Non-Hispanic Black)*Positive (high) | 0.06 | 0.05 | 0.22 | -0.04 | 0.16 | 0.57 |
| Year*Ethnicity (Hispanic)*Positive (no mum and/or dad) | 0.02 | 0.04 | 0.62 | -0.06 | 0.10 | 0.87 |
| Year*Ethnicity (Non-Hispanic Asian)*Positive (no mum and/or dad) | -0.01 | 0.06 | 0.88 | -0.12 | 0.10 | 0.95 |
| Year*Ethnicity (Non-Hispanic Black)*Positive (no mum and/or dad) | 0.05 | 0.05 | 0.26 | -0.04 | 0.15 | 0.65 |
| **Negative experiences with family** |  |  |  |  |  |  |
| Intercept | 2.45 | 0.30 | 0.00 | 1.85 | 3.05 | 0.00 |
| Year | 0.33 | 0.01 | 0.00 | 0.32 | 0.34 | 0.00 |
| Ethnicity (Hispanic) | -0.10 | 0.11 | 0.37 | -0.30 | 0.11 | 0.78 |
| Ethnicity (Non-Hispanic Asian) | -0.30 | 0.14 | 0.03 | -0.58 | -0.02 | 0.15 |
| Ethnicity (Non-Hispanic Black) | -0.03 | 0.11 | 0.81 | -0.25 | 0.20 | 0.92 |
| Negative (medium) | 0.04 | 0.09 | 0.69 | -0.15 | 0.22 | 0.87 |
| Negative (high) | 0.02 | 0.09 | 0.79 | -0.16 | 0.21 | 0.92 |
| Negative (no mum and/or dad) | -0.19 | 0.08 | 0.02 | -0.35 | -0.03 | 0.11 |
| Year*Ethnicity (Hispanic) | 0.03 | 0.01 | 0.01 | 0.01 | 0.06 | 0.05 |
| Year*Ethnicity (Non-Hispanic Asian) | -0.02 | 0.02 | 0.24 | -0.05 | 0.01 | 0.61 |
| Year*Ethnicity (Non-Hispanic Black) | 0.06 | 0.01 | 0.00 | 0.03 | 0.08 | 0.00 |
| Year*Negative (medium) | 0.00 | 0.01 | 0.68 | -0.03 | 0.02 | 0.87 |
| Year*Negative (high) | 0.01 | 0.01 | 0.36 | -0.01 | 0.03 | 0.78 |
| Year*Negative (no mum and/or dad) | 0.01 | 0.01 | 0.11 | 0.00 | 0.03 | 0.32 |
| Ethnicity (Hispanic)*Negative (medium) | -0.03 | 0.20 | 0.88 | -0.42 | 0.36 | 0.95 |
| Ethnicity (Non-Hispanic Asian)*Negative (medium) | 0.23 | 0.28 | 0.42 | -0.33 | 0.78 | 0.81 |
| Ethnicity (Non-Hispanic Black)*Negative (medium) | 0.00 | 0.21 | 0.98 | -0.41 | 0.42 | 0.99 |
| Ethnicity (Hispanic)*Negative (high) | 0.02 | 0.21 | 0.94 | -0.40 | 0.43 | 0.97 |
| Ethnicity (Non-Hispanic Asian)*Negative (high) | 0.22 | 0.31 | 0.47 | -0.38 | 0.83 | 0.83 |
| Ethnicity (Non-Hispanic Black)*Negative (high) | -0.19 | 0.26 | 0.46 | -0.71 | 0.32 | 0.83 |
| Ethnicity (Hispanic)*Negative (no mum and/or dad) | 0.07 | 0.15 | 0.66 | -0.23 | 0.37 | 0.87 |
| Ethnicity (Non-Hispanic Asian)*Negative (no mum and/or dad) | 0.20 | 0.27 | 0.47 | -0.34 | 0.73 | 0.83 |
| Ethnicity (Non-Hispanic Black)*Negative (no mum and/or dad) | -0.05 | 0.14 | 0.71 | -0.34 | 0.23 | 0.88 |
| Year*Ethnicity (Hispanic)*Negative (medium) | 0.02 | 0.02 | 0.48 | -0.03 | 0.06 | 0.84 |
| Year*Ethnicity (Non-Hispanic Asian)*Negative (medium) | -0.01 | 0.03 | 0.80 | -0.07 | 0.06 | 0.92 |
| Year*Ethnicity (Non-Hispanic Black)*Negative (medium) | -0.01 | 0.02 | 0.68 | -0.06 | 0.04 | 0.87 |
| Year*Ethnicity (Hispanic)*Negative (high) | -0.01 | 0.03 | 0.67 | -0.06 | 0.04 | 0.87 |
| Year*Ethnicity (Non-Hispanic Asian)*Negative (high) | -0.02 | 0.04 | 0.65 | -0.09 | 0.05 | 0.87 |
| Year*Ethnicity (Non-Hispanic Black)*Negative (high) | 0.00 | 0.03 | 0.87 | -0.05 | 0.06 | 0.95 |
| Year*Ethnicity (Hispanic)*Negative (no mum and/or dad) | 0.00 | 0.02 | 0.80 | -0.04 | 0.03 | 0.92 |
| Year*Ethnicity (Non-Hispanic Asian)*Negative (no mum and/or dad) | -0.03 | 0.03 | 0.33 | -0.09 | 0.03 | 0.76 |
| Year*Ethnicity (Non-Hispanic Black)*Negative (no mum and/or dad) | -0.01 | 0.02 | 0.58 | -0.04 | 0.02 | 0.87 |

*Note. This table represents findings from linear mixed effect models controlling for age, gender, baseline BMI, neighbourhood disadvantage, parental education and occupation, and self-rated health.* **FDR stands for false discovery rate adjustment, using the Benjamini Hochberg procedure.*

## Supplementary index 10: Results for growth curve model by age.

| **Term** | **Estimate** | **Std error** | **P value** | **2.5 %** | **97.5 %** | **FDR P value** |
| --- | --- | --- | --- | --- | --- | --- |
| **Social contact with family** |  |  |  |  |  |  |
| Intercept | 0.67 | 0.28 | 0.02 | 0.12 | 1.22 | 0.06 |
| Year | 0.38 | 0.03 | 0.00 | 0.32 | 0.43 | 0.00 |
| Age (15-18) | -0.31 | 0.24 | 0.20 | -0.80 | 0.17 | 0.39 |
| Social contact (medium) | -0.06 | 0.22 | 0.80 | -0.49 | 0.38 | 0.87 |
| Social contact (high) | 0.20 | 0.23 | 0.38 | -0.24 | 0.64 | 0.61 |
| Social contact (no mum and/or dad) | -0.27 | 0.23 | 0.23 | -0.72 | 0.18 | 0.45 |
| Year*Age (15-18) | -0.06 | 0.03 | 0.08 | -0.12 | 0.01 | 0.21 |
| Year*Social contact (medium) | 0.00 | 0.03 | 0.96 | -0.06 | 0.06 | 0.98 |
| Year*Social contact (high) | -0.01 | 0.03 | 0.84 | -0.07 | 0.05 | 0.89 |
| Year*Social contact (no mum and/or dad) | 0.01 | 0.03 | 0.71 | -0.05 | 0.07 | 0.84 |
| Age (15-18)*Social contact (medium) | 0.08 | 0.25 | 0.76 | -0.42 | 0.58 | 0.85 |
| Age (15-18)*Social contact (high) | -0.08 | 0.26 | 0.76 | -0.59 | 0.43 | 0.85 |
| Age (15-18)*Social contact (no mum and/or dad) | 0.13 | 0.26 | 0.62 | -0.38 | 0.64 | 0.77 |
| Year*Age (15-18)*Social contact (medium) | 0.01 | 0.03 | 0.76 | -0.06 | 0.08 | 0.85 |
| Year*Age (15-18)*Social contact (high) | 0.02 | 0.03 | 0.49 | -0.04 | 0.09 | 0.70 |
| Year*Age (15-18)*Social contact (no mum and/or dad) | 0.03 | 0.03 | 0.45 | -0.04 | 0.09 | 0.63 |
| **Social contact with friends** |  |  |  |  |  |  |
| Intercept | 0.40 | 0.22 | 0.07 | -0.04 | 0.84 | 0.18 |
| Year | 0.39 | 0.02 | 0.00 | 0.36 | 0.43 | 0.00 |
| Age (15-18) | -0.13 | 0.17 | 0.45 | -0.45 | 0.20 | 0.64 |
| Social contact (medium) | 0.24 | 0.14 | 0.07 | -0.02 | 0.51 | 0.19 |
| Social contact (high) | 0.05 | 0.15 | 0.74 | -0.24 | 0.34 | 0.85 |
| Year*Age (15-18) | -0.02 | 0.02 | 0.41 | -0.06 | 0.03 | 0.61 |
| Year*Social contact (medium) | -0.03 | 0.02 | 0.14 | -0.06 | 0.01 | 0.31 |
| Year*Social contact (high) | -0.01 | 0.02 | 0.55 | -0.05 | 0.03 | 0.75 |
| Age (15-18)*Social contact (medium) | -0.27 | 0.18 | 0.12 | -0.62 | 0.07 | 0.28 |
| Age (15-18)*Social contact (high) | -0.04 | 0.19 | 0.83 | -0.41 | 0.33 | 0.89 |
| Year*Age (15-18)*Social contact (medium) | -0.01 | 0.02 | 0.57 | -0.06 | 0.03 | 0.76 |
| Year*Age (15-18)*Social contact (high) | -0.03 | 0.03 | 0.19 | -0.08 | 0.02 | 0.38 |
| **Relationship status** |  |  |  |  |  |  |
| Intercept | 0.54 | 0.21 | 0.01 | 0.12 | 0.96 | 0.05 |
| Year | 0.38 | 0.01 | 0.00 | 0.35 | 0.40 | 0.00 |
| Age (15-18) | -0.26 | 0.10 | 0.01 | -0.47 | -0.06 | 0.05 |
| Relationship status (single) | 0.01 | 0.11 | 0.94 | -0.21 | 0.22 | 0.97 |
| Year*Age (15-18) | -0.03 | 0.01 | 0.02 | -0.06 | -0.01 | 0.06 |
| Year*Relationship status (single) | 0.00 | 0.01 | 0.94 | -0.03 | 0.03 | 0.97 |
| Age (15-18)*Relationship status (single) | -0.02 | 0.13 | 0.87 | -0.27 | 0.23 | 0.92 |
| Year*Age (15-18)*Relationship status (single) | -0.01 | 0.02 | 0.33 | -0.04 | 0.02 | 0.60 |
| **Loneliness** |  |  |  |  |  |  |
| Intercept | 0.59 | 0.19 | 0.00 | 0.22 | 0.96 | 0.01 |
| Year | 0.38 | 0.01 | 0.00 | 0.36 | 0.39 | 0.00 |
| Age (15-18) | -0.29 | 0.06 | 0.00 | -0.42 | -0.17 | 0.00 |
| Loneliness (medium) | -0.25 | 0.10 | 0.02 | -0.45 | -0.05 | 0.06 |
| Loneliness (high) | -0.17 | 0.19 | 0.36 | -0.53 | 0.19 | 0.61 |
| Year*Age (15-18) | -0.05 | 0.01 | 0.00 | -0.06 | -0.03 | 0.00 |
| Year*Loneliness (medium) | 0.00 | 0.01 | 0.83 | -0.02 | 0.03 | 0.89 |
| Year*Loneliness (high) | -0.01 | 0.02 | 0.77 | -0.05 | 0.04 | 0.85 |
| Age (15-18)*Loneliness (medium) | 0.10 | 0.12 | 0.42 | -0.14 | 0.33 | 0.61 |
| Age (15-18)*Loneliness (high) | 0.11 | 0.20 | 0.57 | -0.28 | 0.51 | 0.76 |
| Year*Age (15-18)*Loneliness (medium) | 0.01 | 0.02 | 0.39 | -0.02 | 0.05 | 0.61 |
| Year*Age (15-18)*Loneliness (high) | 0.02 | 0.03 | 0.35 | -0.03 | 0.07 | 0.61 |
| **Perceived support from family** |  |  |  |  |  |  |
| Intercept | 0.52 | 0.35 | 0.13 | -0.16 | 1.20 | 0.30 |
| Year | 0.35 | 0.04 | 0.00 | 0.28 | 0.43 | 0.00 |
| Age (15-18) | -0.14 | 0.33 | 0.68 | -0.79 | 0.52 | 0.82 |
| Perceived support (medium) | 0.08 | 0.30 | 0.79 | -0.52 | 0.68 | 0.86 |
| Perceived support (high) | 0.26 | 0.29 | 0.38 | -0.32 | 0.84 | 0.61 |
| Perceived support (no mum and/or dad) | -0.12 | 0.30 | 0.70 | -0.71 | 0.48 | 0.84 |
| Year*Age (15-18) | -0.01 | 0.04 | 0.85 | -0.09 | 0.07 | 0.90 |
| Year*Perceived support (medium) | 0.02 | 0.04 | 0.65 | -0.06 | 0.10 | 0.79 |
| Year*Perceived support (high) | 0.02 | 0.04 | 0.61 | -0.06 | 0.10 | 0.77 |
| Year*Perceived support (no mum and/or dad) | 0.03 | 0.04 | 0.41 | -0.05 | 0.11 | 0.61 |
| Age (15-18)*Perceived support (medium) | -0.15 | 0.36 | 0.68 | -0.85 | 0.55 | 0.82 |
| Age (15-18)*Perceived support (high) | -0.19 | 0.34 | 0.59 | -0.86 | 0.49 | 0.76 |
| Age (15-18)*Perceived support (no mum and/or dad) | -0.05 | 0.34 | 0.88 | -0.72 | 0.62 | 0.93 |
| Year*Age (15-18)*Perceived support (medium) | -0.02 | 0.04 | 0.58 | -0.11 | 0.06 | 0.76 |
| Year*Age (15-18)*Perceived support (high) | -0.04 | 0.04 | 0.34 | -0.13 | 0.04 | 0.61 |
| Year*Age (15-18)*Perceived support (no mum and/or dad) | -0.02 | 0.04 | 0.58 | -0.11 | 0.06 | 0.76 |
| **Received support from family** |  |  |  |  |  |  |
| Intercept | 0.76 | 0.21 | 0.00 | 0.35 | 1.16 | 0.00 |
| Year | 0.37 | 0.01 | 0.00 | 0.35 | 0.39 | 0.00 |
| Age (15-18) | -0.37 | 0.10 | 0.00 | -0.56 | -0.18 | 0.00 |
| Received support (medium) | 0.00 | 0.11 | 0.99 | -0.21 | 0.21 | 0.99 |
| Received support (high) | 0.04 | 0.20 | 0.85 | -0.35 | 0.43 | 0.90 |
| Received support (no mum and/or dad) | -0.33 | 0.11 | 0.00 | -0.54 | -0.12 | 0.01 |
| Year*Age (15-18) | -0.04 | 0.01 | 0.00 | -0.07 | -0.02 | 0.00 |
| Year*Received support (medium) | 0.01 | 0.01 | 0.59 | -0.02 | 0.03 | 0.76 |
| Year*Received support (high) | 0.02 | 0.03 | 0.54 | -0.04 | 0.07 | 0.74 |
| Year*Received support (no mum and/or dad) | 0.02 | 0.01 | 0.15 | -0.01 | 0.04 | 0.32 |
| Age (15-18)*Received support (medium) | 0.06 | 0.13 | 0.65 | -0.20 | 0.32 | 0.79 |
| Age (15-18)*Received support (high) | 0.52 | 0.25 | 0.04 | 0.03 | 1.00 | 0.10 |
| Age (15-18)*Received support (no mum and/or dad) | 0.19 | 0.13 | 0.14 | -0.06 | 0.43 | 0.31 |
| Year*Age (15-18)*Received support (medium) | 0.00 | 0.02 | 0.77 | -0.04 | 0.03 | 0.85 |
| Year*Age (15-18)*Received support (high) | 0.01 | 0.03 | 0.69 | -0.05 | 0.08 | 0.83 |
| Year*Age (15-18)*Received support (no mum and/or dad) | 0.01 | 0.02 | 0.55 | -0.02 | 0.04 | 0.75 |
| **Perceived support from friends** |  |  |  |  |  |  |
| Intercept | 0.05 | 0.32 | 0.88 | -0.58 | 0.67 | 0.93 |
| Year | 0.39 | 0.03 | 0.00 | 0.32 | 0.45 | 0.00 |
| Age (15-18) | 0.11 | 0.30 | 0.71 | -0.49 | 0.71 | 0.84 |
| Perceived support (medium) | 0.48 | 0.26 | 0.07 | -0.03 | 1.00 | 0.17 |
| Perceived support (high) | 0.56 | 0.27 | 0.04 | 0.03 | 1.08 | 0.10 |
| Year*Age (15-18) | 0.00 | 0.04 | 0.93 | -0.08 | 0.07 | 0.97 |
| Year*Perceived support (medium) | 0.00 | 0.03 | 0.94 | -0.07 | 0.06 | 0.97 |
| Year*Perceived support (high) | -0.02 | 0.03 | 0.62 | -0.08 | 0.05 | 0.77 |
| Age (15-18)*Perceived support (medium) | -0.44 | 0.31 | 0.17 | -1.05 | 0.18 | 0.35 |
| Age (15-18)*Perceived support (high) | -0.36 | 0.32 | 0.25 | -0.98 | 0.26 | 0.49 |
| Year*Age (15-18)*Perceived support (medium) | -0.04 | 0.04 | 0.35 | -0.12 | 0.04 | 0.61 |
| Year*Age (15-18)*Perceived support (high) | -0.04 | 0.04 | 0.37 | -0.11 | 0.04 | 0.61 |
| **Received support from friends** |  |  |  |  |  |  |
| Intercept | 0.60 | 0.19 | 0.00 | 0.22 | 0.98 | 0.01 |
| Year | 0.38 | 0.01 | 0.00 | 0.36 | 0.40 | 0.00 |
| Age (15-18) | -0.39 | 0.09 | 0.00 | -0.57 | -0.22 | 0.00 |
| Received support (medium) | -0.06 | 0.10 | 0.53 | -0.25 | 0.13 | 0.74 |
| Received support (high) | -0.09 | 0.11 | 0.42 | -0.31 | 0.13 | 0.61 |
| Year*Age (15-18) | -0.04 | 0.01 | 0.00 | -0.06 | -0.02 | 0.01 |
| Year*Received support (medium) | -0.01 | 0.01 | 0.61 | -0.03 | 0.02 | 0.77 |
| Year*Received support (high) | 0.00 | 0.01 | 0.79 | -0.03 | 0.02 | 0.86 |
| Age (15-18)*Received support (medium) | 0.18 | 0.12 | 0.13 | -0.05 | 0.41 | 0.30 |
| Age (15-18)*Received support (high) | 0.18 | 0.14 | 0.19 | -0.09 | 0.44 | 0.38 |
| Year*Age (15-18)*Received support (medium) | 0.00 | 0.01 | 0.81 | -0.03 | 0.03 | 0.88 |
| Year*Age (15-18)*Received support (high) | 0.00 | 0.02 | 0.97 | -0.03 | 0.03 | 0.98 |
| **Positive experiences with family** |  |  |  |  |  |  |
| Intercept | 0.62 | 0.32 | 0.05 | -0.01 | 1.25 | 0.14 |
| Year | 0.33 | 0.03 | 0.00 | 0.26 | 0.40 | 0.00 |
| Age (15-18) | -0.45 | 0.28 | 0.11 | -1.00 | 0.11 | 0.27 |
| Positive (medium) | 0.01 | 0.28 | 0.98 | -0.55 | 0.56 | 0.99 |
| Positive (high) | 0.16 | 0.26 | 0.54 | -0.35 | 0.67 | 0.74 |
| Positive (no mum and/or dad) | -0.21 | 0.27 | 0.44 | -0.74 | 0.32 | 0.63 |
| Year*Age (15-18) | 0.00 | 0.04 | 0.94 | -0.07 | 0.08 | 0.97 |
| Year*Positive (medium) | 0.04 | 0.04 | 0.30 | -0.03 | 0.11 | 0.55 |
| Year*Positive (high) | 0.05 | 0.03 | 0.19 | -0.02 | 0.11 | 0.38 |
| Year*Positive (no mum and/or dad) | 0.06 | 0.04 | 0.11 | -0.01 | 0.13 | 0.26 |
| Age (15-18)*Positive (medium) | 0.13 | 0.31 | 0.67 | -0.48 | 0.74 | 0.82 |
| Age (15-18)*Positive (high) | 0.18 | 0.29 | 0.54 | -0.39 | 0.75 | 0.74 |
| Age (15-18)*Positive (no mum and/or dad) | 0.26 | 0.30 | 0.37 | -0.32 | 0.84 | 0.61 |
| Year*Age (15-18)*Positive (medium) | -0.04 | 0.04 | 0.32 | -0.12 | 0.04 | 0.58 |
| Year*Age (15-18)*Positive (high) | -0.05 | 0.04 | 0.20 | -0.12 | 0.03 | 0.38 |
| Year*Age (15-18)*Positive (no mum and/or dad) | -0.03 | 0.04 | 0.36 | -0.11 | 0.04 | 0.61 |
| **Negative experiences with family** |  |  |  |  |  |  |
| Intercept | 0.80 | 0.20 | 0.00 | 0.40 | 1.20 | 0.00 |
| Year | 0.37 | 0.01 | 0.00 | 0.35 | 0.39 | 0.00 |
| Age (15-18) | -0.40 | 0.08 | 0.00 | -0.56 | -0.25 | 0.00 |
| Negative (medium) negative | -0.07 | 0.13 | 0.58 | -0.34 | 0.19 | 0.76 |
| Negative (high) negative | -0.15 | 0.13 | 0.24 | -0.41 | 0.10 | 0.46 |
| Negative (no mum and/or dad) | -0.38 | 0.10 | 0.00 | -0.57 | -0.18 | 0.00 |
| Year*Age (15-18) | -0.04 | 0.01 | 0.00 | -0.06 | -0.02 | 0.00 |
| Year*Negative (medium) negative | 0.00 | 0.02 | 0.92 | -0.03 | 0.04 | 0.96 |
| Year*Negative (high) negative | 0.00 | 0.02 | 0.83 | -0.04 | 0.03 | 0.89 |
| Year*Negative (no mum and/or dad) | 0.01 | 0.01 | 0.22 | -0.01 | 0.04 | 0.42 |
| Age (15-18)*Negative (medium) negative | 0.17 | 0.15 | 0.27 | -0.13 | 0.47 | 0.50 |
| Age (15-18)*Negative (high) negative | 0.27 | 0.16 | 0.10 | -0.05 | 0.58 | 0.24 |
| Age (15-18)*Negative (no mum and/or dad) | 0.22 | 0.12 | 0.06 | -0.01 | 0.45 | 0.17 |
| Year*Age (15-18)*Negative (medium) negative | 0.00 | 0.02 | 0.83 | -0.04 | 0.03 | 0.89 |
| Year*Age (15-18)*Negative (high) negative | 0.01 | 0.02 | 0.53 | -0.03 | 0.05 | 0.74 |
| Year*Age (15-18)*Negative (no mum and/or dad) | 0.01 | 0.01 | 0.40 | -0.02 | 0.04 | 0.61 |

*Note. This table represents findings from linear mixed effect models controlling for gender, ethnicity, baseline BMI, neighbourhood disadvantage, parental education and occupation, and self-rated health.* **FDR stands for false discovery rate adjustment, using the Benjamini Hochberg procedure.*

## Supplementary Index 11: Unadjusted growth curve models.

| **Term** | **Estimate** | **Std error** | **P value** | **2.5 %** | **97.5 %** | **P value FDR** |
| --- | --- | --- | --- | --- | --- | --- |
| **Social contact with family** |  |  |  |  |  |  |
| Intercept | 23.76 | 0.18 | 0.00 | 23.41 | 24.10 | 0.00 |
| Year | 0.33 | 0.01 | 0.00 | 0.31 | 0.35 | 0.00 |
| Social contact (medium) | -0.37 | 0.19 | 0.05 | -0.73 | 0.00 | 0.08 |
| Social contact (high) | -0.15 | 0.19 | 0.44 | -0.52 | 0.23 | 0.56 |
| Social contact (no mum and/or dad) | 0.10 | 0.19 | 0.60 | -0.27 | 0.47 | 0.68 |
| School ID | 0.00 | 0.00 | 0.00 | 0.00 | 0.00 | 0.00 |
| Year*Social contact (medium) | 0.01 | 0.01 | 0.35 | -0.01 | 0.03 | 0.46 |
| Year*Social contact (high) | 0.02 | 0.01 | 0.17 | -0.01 | 0.04 | 0.24 |
| Year*Social contact (no mum and/or dad) | 0.03 | 0.01 | 0.01 | 0.01 | 0.06 | 0.02 |
| **Social contact with friends** |  |  |  |  |  |  |
| Intercept | 24.31 | 0.14 | 0.00 | 24.03 | 24.59 | 0.00 |
| Year | 0.38 | 0.01 | 0.00 | 0.36 | 0.40 | 0.00 |
| Social contact (medium) | -0.71 | 0.15 | 0.00 | -1.00 | -0.42 | 0.00 |
| Social contact (high) | -0.82 | 0.15 | 0.00 | -1.12 | -0.52 | 0.00 |
| School ID | 0.00 | 0.00 | 0.00 | 0.00 | 0.00 | 0.00 |
| Year*Social contact (medium) | -0.04 | 0.01 | 0.00 | -0.05 | -0.02 | 0.00 |
| Year*Social contact (high) | -0.04 | 0.01 | 0.00 | -0.06 | -0.02 | 0.00 |
| **Relationship status** |  |  |  |  |  |  |
| Intercept | 23.58 | 0.08 | 0.00 | 23.43 | 23.73 | 0.00 |
| Year | 0.35 | 0.01 | 0.00 | 0.34 | 0.36 | 0.00 |
| Relationship status (single) | 0.06 | 0.09 | 0.47 | -0.11 | 0.23 | 0.57 |
| School ID | 0.00 | 0.00 | 0.00 | 0.00 | 0.00 | 0.00 |
| Year*Relationship status (single) | 0.00 | 0.01 | 0.48 | -0.02 | 0.01 | 0.58 |
| **Loneliness** |  |  |  |  |  |  |
| Intercept | 23.52 | 0.06 | 0.00 | 23.39 | 23.64 | 0.00 |
| Year | 0.35 | 0.00 | 0.00 | 0.34 | 0.36 | 0.00 |
| Loneliness (medium) | 0.18 | 0.10 | 0.06 | -0.01 | 0.36 | 0.10 |
| Loneliness (high) | 0.59 | 0.16 | 0.00 | 0.29 | 0.90 | 0.00 |
| School ID | 0.00 | 0.00 | 0.00 | 0.00 | 0.00 | 0.00 |
| Year*Loneliness (medium) | 0.01 | 0.01 | 0.19 | 0.00 | 0.02 | 0.26 |
| Year*Loneliness (high) | 0.01 | 0.01 | 0.53 | -0.01 | 0.03 | 0.62 |
| **Perceived support from family** |  |  |  |  |  |  |
| Intercept | 23.84 | 0.27 | 0.00 | 23.31 | 24.38 | 0.00 |
| Year | 0.35 | 0.02 | 0.00 | 0.31 | 0.39 | 0.00 |
| Perceived support (medium) | -0.21 | 0.29 | 0.47 | -0.77 | 0.35 | 0.57 |
| Perceived support (high) | -0.41 | 0.28 | 0.14 | -0.96 | 0.13 | 0.20 |
| Perceived support (no mum and/or dad) | 0.02 | 0.28 | 0.94 | -0.53 | 0.57 | 0.97 |
| School ID | 0.00 | 0.00 | 0.00 | 0.00 | 0.00 | 0.00 |
| Year*Perceived support (medium) | 0.00 | 0.02 | 0.95 | -0.04 | 0.04 | 0.97 |
| Year*Perceived support (high) | -0.01 | 0.02 | 0.70 | -0.05 | 0.03 | 0.75 |
| Year*Perceived support (no mum and/or dad) | 0.02 | 0.02 | 0.42 | -0.02 | 0.05 | 0.54 |
| **Received support from family** |  |  |  |  |  |  |
| Intercept | 23.41 | 0.08 | 0.00 | 23.26 | 23.56 | 0.00 |
| Year | 0.34 | 0.00 | 0.00 | 0.33 | 0.35 | 0.00 |
| Received support (medium) | 0.18 | 0.10 | 0.09 | -0.03 | 0.38 | 0.13 |
| Received support (high) | 0.34 | 0.23 | 0.13 | -0.10 | 0.79 | 0.19 |
| Received support (no mum and/or dad) | 0.45 | 0.10 | 0.00 | 0.24 | 0.65 | 0.00 |
| School ID | 0.00 | 0.00 | 0.00 | 0.00 | 0.00 | 0.00 |
| Year*Received support (medium) | 0.00 | 0.01 | 0.77 | -0.01 | 0.01 | 0.81 |
| Year*Received support (high) | 0.03 | 0.01 | 0.05 | 0.00 | 0.05 | 0.08 |
| Year*Received support (no mum and/or dad) | 0.02 | 0.01 | 0.00 | 0.01 | 0.04 | 0.00 |
| **Perceived support from friends** |  |  |  |  |  |  |
| Intercept | 24.23 | 0.25 | 0.00 | 23.73 | 24.73 | 0.00 |
| Year | 0.38 | 0.02 | 0.00 | 0.35 | 0.42 | 0.00 |
| Perceived support (medium) | -0.53 | 0.26 | 0.04 | -1.04 | -0.03 | 0.07 |
| Perceived support (high) | -0.75 | 0.26 | 0.00 | -1.26 | -0.24 | 0.01 |
| School ID | 0.00 | 0.00 | 0.00 | 0.00 | 0.00 | 0.00 |
| Year*Perceived support (medium) | -0.03 | 0.02 | 0.12 | -0.06 | 0.01 | 0.18 |
| Year*Perceived support (high) | -0.04 | 0.02 | 0.02 | -0.08 | -0.01 | 0.04 |
| **Received support from friends** |  |  |  |  |  |  |
| Intercept | 23.59 | 0.08 | 0.00 | 23.43 | 23.75 | 0.00 |
| Year | 0.36 | 0.01 | 0.00 | 0.35 | 0.37 | 0.00 |
| Received support (medium) | 0.10 | 0.10 | 0.35 | -0.11 | 0.30 | 0.46 |
| Received support (high) | -0.02 | 0.10 | 0.87 | -0.22 | 0.18 | 0.90 |
| School ID | 0.00 | 0.00 | 0.00 | 0.00 | 0.00 | 0.00 |
| Year*Received support (medium) | -0.01 | 0.01 | 0.06 | -0.03 | 0.00 | 0.10 |
| Year*Received support (high) | -0.01 | 0.01 | 0.09 | -0.02 | 0.00 | 0.13 |
| **Positive experiences with family** |  |  |  |  |  |  |
| Intercept | 0.29 | 0.17 | 0.09 | -0.04 | 0.62 | 0.13 |
| Year | 0.33 | 0.01 | 0.00 | 0.30 | 0.36 | 0.00 |
| Positive (medium) | 0.07 | 0.13 | 0.57 | -0.18 | 0.32 | 0.66 |
| Positive (high) | 0.26 | 0.12 | 0.03 | 0.02 | 0.49 | 0.06 |
| Positive (no mum and/or dad) | 0.05 | 0.12 | 0.68 | -0.19 | 0.29 | 0.74 |
| School ID | 0.00 | 0.00 | 1.00 | 0.00 | 0.00 | 1.00 |
| baseline_bmi | 1.02 | 0.01 | 0.00 | 1.01 | 1.03 | 0.00 |
| Year*Positive (medium) | 0.01 | 0.02 | 0.67 | -0.03 | 0.04 | 0.74 |
| Year*Positive (high) | 0.01 | 0.02 | 0.42 | -0.02 | 0.04 | 0.54 |
| Year*Positive (no mum and/or dad) | 0.03 | 0.02 | 0.04 | 0.00 | 0.06 | 0.07 |
| **Negative experiences with family** |  |  |  |  |  |  |
| Intercept | 23.41 | 0.07 | 0.00 | 23.27 | 23.56 | 0.00 |
| Year | 0.34 | 0.00 | 0.00 | 0.33 | 0.35 | 0.00 |
| Negative (medium) | 0.25 | 0.13 | 0.06 | -0.01 | 0.50 | 0.10 |
| Negative (high) | 0.21 | 0.14 | 0.13 | -0.06 | 0.47 | 0.19 |
| Negative (no mum and/or dad) | 0.44 | 0.10 | 0.00 | 0.24 | 0.63 | 0.00 |
| School ID | 0.00 | 0.00 | 0.00 | 0.00 | 0.00 | 0.00 |
| Year*Negative (medium) | 0.00 | 0.01 | 0.69 | -0.02 | 0.01 | 0.75 |
| Year*Negative (high) | 0.00 | 0.01 | 0.56 | -0.01 | 0.02 | 0.65 |
| Year*Negative (no mum and/or dad) | 0.02 | 0.01 | 0.00 | 0.01 | 0.04 | 0.01 |

## Supplementary Index 12: Growth curve models unadjusted for baseline BMI.

| **Term** | **Estimate** | **Std error** | **P value** | **2.5 %** | **97.5 %** | **P value FDR** |
| --- | --- | --- | --- | --- | --- | --- |
| **Social contact with family** |  |  |  |  |  |  |
| Intercept | 17.24 | 0.54 | 0.00 | 16.18 | 18.29 | 0.00 |
| Year | 0.33 | 0.01 | 0.00 | 0.31 | 0.35 | 0.00 |
| Social contact (medium) | -0.15 | 0.18 | 0.42 | -0.50 | 0.21 | 0.45 |
| Social contact (high) | 0.18 | 0.19 | 0.35 | -0.19 | 0.55 | 0.38 |
| Social contact (no mum and/or dad) | -0.22 | 0.19 | 0.24 | -0.59 | 0.15 | 0.27 |
| Year*Social contact (medium) | 0.01 | 0.01 | 0.34 | -0.01 | 0.03 | 0.37 |
| Year*Social contact (high) | 0.02 | 0.01 | 0.17 | -0.01 | 0.04 | 0.20 |
| Year*Social contact (no mum and/or dad) | 0.03 | 0.01 | 0.01 | 0.01 | 0.06 | 0.01 |
| **Social contact with friends** |  |  |  |  |  |  |
| Intercept | 17.65 | 0.50 | 0.00 | 16.66 | 18.64 | 0.00 |
| Year | 0.38 | 0.01 | 0.00 | 0.36 | 0.40 | 0.00 |
| Social contact (medium) | -0.51 | 0.14 | 0.00 | -0.79 | -0.23 | 0.00 |
| Social contact (high) | -0.71 | 0.15 | 0.00 | -1.00 | -0.42 | 0.00 |
| Year*Social contact (medium) | -0.04 | 0.01 | 0.00 | -0.05 | -0.02 | 0.00 |
| Year*Social contact (high) | -0.04 | 0.01 | 0.00 | -0.06 | -0.02 | 0.00 |
| **Relationship status** |  |  |  |  |  |  |
| Intercept | 16.41 | 0.52 | 0.00 | 15.39 | 17.43 | 0.00 |
| Year | 0.35 | 0.01 | 0.00 | 0.34 | 0.36 | 0.00 |
| Relationship status (single) | 0.43 | 0.09 | 0.00 | 0.26 | 0.60 | 0.00 |
| Year*Relationship status (single) | 0.00 | 0.01 | 0.47 | -0.02 | 0.01 | 0.49 |
| **Loneliness** |  |  |  |  |  |  |
| Intercept | 17.10 | 0.49 | 0.00 | 16.13 | 18.07 | 0.00 |
| Year | 0.35 | 0.00 | 0.00 | 0.34 | 0.36 | 0.00 |
| Loneliness (medium) | -0.16 | 0.09 | 0.09 | -0.34 | 0.03 | 0.12 |
| Loneliness (high) | -0.14 | 0.15 | 0.34 | -0.44 | 0.16 | 0.37 |
| Year*Loneliness (medium) | 0.01 | 0.01 | 0.18 | 0.00 | 0.02 | 0.22 |
| Year*Loneliness (high) | 0.01 | 0.01 | 0.51 | -0.01 | 0.03 | 0.53 |
| **Perceived support from family** |  |  |  |  |  |  |
| Intercept | 17.01 | 0.57 | 0.00 | 15.89 | 18.13 | 0.00 |
| Year | 0.35 | 0.02 | 0.00 | 0.31 | 0.39 | 0.00 |
| Perceived support (medium) | 0.06 | 0.28 | 0.82 | -0.48 | 0.61 | 0.84 |
| Perceived support (high) | 0.32 | 0.27 | 0.24 | -0.21 | 0.86 | 0.26 |
| Perceived support (no mum and/or dad) | 0.03 | 0.28 | 0.90 | -0.51 | 0.58 | 0.92 |
| Year*Perceived support (medium) | 0.00 | 0.02 | 0.95 | -0.04 | 0.04 | 0.96 |
| Year*Perceived support (high) | -0.01 | 0.02 | 0.69 | -0.05 | 0.03 | 0.72 |
| Year*Perceived support (no mum and/or dad) | 0.02 | 0.02 | 0.41 | -0.02 | 0.05 | 0.44 |
| **Received support from family** |  |  |  |  |  |  |
| Intercept | 17.25 | 0.50 | 0.00 | 16.26 | 18.24 | 0.00 |
| Year | 0.34 | 0.00 | 0.00 | 0.33 | 0.35 | 0.00 |
| Received support (medium) | 0.18 | 0.10 | 0.07 | -0.02 | 0.38 | 0.10 |
| Received support (high) | 0.60 | 0.22 | 0.01 | 0.17 | 1.03 | 0.01 |
| Received support (no mum and/or dad) | -0.10 | 0.11 | 0.35 | -0.32 | 0.12 | 0.38 |
| Year*Received support (medium) | 0.00 | 0.01 | 0.78 | -0.01 | 0.01 | 0.80 |
| Year*Received support (high) | 0.03 | 0.01 | 0.05 | 0.00 | 0.05 | 0.07 |
| Year*Received support (no mum and/or dad) | 0.02 | 0.01 | 0.00 | 0.01 | 0.04 | 0.00 |
| **Perceived support from friends** |  |  |  |  |  |  |
| Intercept | 17.12 | 0.55 | 0.00 | 16.05 | 18.19 | 0.00 |
| Year | 0.38 | 0.02 | 0.00 | 0.35 | 0.42 | 0.00 |
| Perceived support (medium) | 0.00 | 0.26 | 1.00 | -0.50 | 0.50 | 1.00 |
| Perceived support (high) | 0.08 | 0.26 | 0.77 | -0.43 | 0.58 | 0.79 |
| Year*Perceived support (medium) | -0.03 | 0.02 | 0.12 | -0.06 | 0.01 | 0.16 |
| Year*Perceived support (high) | -0.04 | 0.02 | 0.02 | -0.08 | -0.01 | 0.03 |
| **Received support from friends** |  |  |  |  |  |  |
| Intercept | 17.04 | 0.50 | 0.00 | 16.07 | 18.01 | 0.00 |
| Year | 0.36 | 0.01 | 0.00 | 0.35 | 0.37 | 0.00 |
| Received support (medium) | 0.00 | 0.10 | 0.97 | -0.21 | 0.20 | 0.98 |
| Received support (high) | -0.16 | 0.11 | 0.12 | -0.37 | 0.04 | 0.16 |
| Year*Received support (medium) | -0.01 | 0.01 | 0.06 | -0.03 | 0.00 | 0.09 |
| Year*Received support (high) | -0.01 | 0.01 | 0.09 | -0.02 | 0.00 | 0.12 |
| **Positive experiences with family** |  |  |  |  |  |  |
| Intercept | 16.12 | 0.56 | 0.00 | 15.02 | 17.21 | 0.00 |
| Year | 0.33 | 0.01 | 0.00 | 0.31 | 0.36 | 0.00 |
| Positive (medium) | 0.41 | 0.23 | 0.07 | -0.04 | 0.85 | 0.10 |
| Positive (high) | 1.04 | 0.22 | 0.00 | 0.62 | 1.47 | 0.00 |
| Positive (no mum and/or dad) | 0.59 | 0.22 | 0.01 | 0.15 | 1.03 | 0.01 |
| Year*Positive (medium) | 0.01 | 0.01 | 0.70 | -0.02 | 0.03 | 0.72 |
| Year*Positive (high) | 0.01 | 0.01 | 0.39 | -0.02 | 0.04 | 0.42 |
| Year*Positive (no mum and/or dad) | 0.03 | 0.01 | 0.02 | 0.00 | 0.06 | 0.04 |
| **Negative experiences with family** |  |  |  |  |  |  |
| Intercept | 17.32 | 0.50 | 0.00 | 16.33 | 18.31 | 0.00 |
| Year | 0.34 | 0.00 | 0.00 | 0.33 | 0.35 | 0.00 |
| Negative (medium) | 0.01 | 0.13 | 0.93 | -0.24 | 0.26 | 0.95 |
| Negative (high) | 0.10 | 0.13 | 0.46 | -0.16 | 0.36 | 0.48 |
| Negative (no mum and/or dad) | -0.20 | 0.11 | 0.07 | -0.41 | 0.01 | 0.09 |
| Year*Negative (medium) | 0.00 | 0.01 | 0.69 | -0.02 | 0.01 | 0.71 |
| Year*Negative (high) | 0.00 | 0.01 | 0.56 | -0.01 | 0.02 | 0.58 |
| Year*Negative (no mum and/or dad) | 0.02 | 0.01 | 0.00 | 0.01 | 0.04 | 0.01 |

*Note. This table represents findings from linear mixed effect models controlling for age, gender, ethnicity, neighbourhood disadvantage, parental education and occupation, and self-rated health.*

## Supplementary Index 13: Growth curve models adjusted for depression.

| **Term** | **Estimate** | **Std error** | **P value** | **2.5 %** | **97.5 %** | **P value FDR** |
| --- | --- | --- | --- | --- | --- | --- |
| **Social contact with family** |  |  |  |  |  |  |
| Intercept | 2.34 | 0.31 | 0.00 | 1.72 | 2.95 | 0.00 |
| Year | 0.33 | 0.01 | 0.00 | 0.30 | 0.35 | 0.00 |
| Social contact (medium) | -0.02 | 0.11 | 0.84 | -0.23 | 0.19 | 0.85 |
| Social contact (high) | 0.11 | 0.11 | 0.31 | -0.11 | 0.34 | 0.50 |
| Social contact (no mum and/or dad) | -0.20 | 0.11 | 0.08 | -0.43 | 0.02 | 0.16 |
| CESD total | 0.00 | 0.00 | 0.92 | -0.01 | 0.01 | 0.92 |
| Year*Social contact (medium) | 0.01 | 0.01 | 0.34 | -0.01 | 0.04 | 0.53 |
| Year*Social contact (high) | 0.02 | 0.01 | 0.18 | -0.01 | 0.05 | 0.32 |
| Year*Social contact (no mum and/or dad) | 0.04 | 0.01 | 0.01 | 0.01 | 0.06 | 0.04 |
| **Social contact with friends** |  |  |  |  |  |  |
| Intercept | 2.21 | 0.27 | 0.00 | 1.68 | 2.74 | 0.00 |
| Year | 0.38 | 0.01 | 0.00 | 0.36 | 0.40 | 0.00 |
| Social contact (medium) | 0.07 | 0.08 | 0.41 | -0.09 | 0.23 | 0.58 |
| Social contact (high) | 0.04 | 0.08 | 0.67 | -0.13 | 0.20 | 0.74 |
| CESD total | 0.00 | 0.00 | 0.83 | -0.01 | 0.00 | 0.85 |
| Year*Social contact (medium) | -0.04 | 0.01 | 0.00 | -0.06 | -0.02 | 0.00 |
| Year*Social contact (high) | -0.04 | 0.01 | 0.00 | -0.06 | -0.02 | 0.00 |
| **Relationship status** |  |  |  |  |  |  |
| Intercept | 2.39 | 0.29 | 0.00 | 1.82 | 2.95 | 0.00 |
| Year | 0.35 | 0.01 | 0.00 | 0.34 | 0.36 | 0.00 |
| Relationship status (single) | -0.07 | 0.05 | 0.18 | -0.17 | 0.03 | 0.32 |
| CESD total | 0.00 | 0.00 | 0.75 | -0.01 | 0.00 | 0.79 |
| Year*Relationship status (single) | 0.00 | 0.01 | 0.45 | -0.02 | 0.01 | 0.59 |
| **Loneliness** |  |  |  |  |  |  |
| Intercept | 2.20 | 0.29 | 0.00 | 1.64 | 2.77 | 0.00 |
| Year | 0.35 | 0.00 | 0.00 | 0.34 | 0.36 | 0.00 |
| Loneliness (medium) | -0.18 | 0.06 | 0.00 | -0.29 | -0.06 | 0.01 |
| Loneliness (high) | -0.10 | 0.10 | 0.33 | -0.30 | 0.10 | 0.53 |
| CESD total | 0.00 | 0.00 | 0.36 | 0.00 | 0.01 | 0.55 |
| Year*Loneliness (medium) | 0.01 | 0.01 | 0.20 | -0.01 | 0.02 | 0.34 |
| Year*Loneliness (high) | 0.01 | 0.01 | 0.58 | -0.02 | 0.03 | 0.70 |
| **Perceived support from family** |  |  |  |  |  |  |
| Intercept | 2.32 | 0.33 | 0.00 | 1.66 | 2.97 | 0.00 |
| Year | 0.35 | 0.02 | 0.00 | 0.31 | 0.39 | 0.00 |
| Perceived support (medium) | -0.02 | 0.16 | 0.90 | -0.33 | 0.29 | 0.91 |
| Perceived support (high) | 0.12 | 0.16 | 0.45 | -0.19 | 0.43 | 0.59 |
| Perceived support (no mum and/or dad) | -0.16 | 0.16 | 0.33 | -0.47 | 0.16 | 0.53 |
| CESD total | 0.00 | 0.00 | 0.75 | 0.00 | 0.01 | 0.79 |
| Year*Perceived support (medium) | 0.00 | 0.02 | 0.97 | -0.04 | 0.04 | 0.97 |
| Year*Perceived support (high) | -0.01 | 0.02 | 0.71 | -0.05 | 0.03 | 0.77 |
| Year*Perceived support (no mum and/or dad) | 0.02 | 0.02 | 0.44 | -0.03 | 0.06 | 0.58 |
| **Received support from family** |  |  |  |  |  |  |
| Intercept | 2.39 | 0.28 | 0.00 | 1.83 | 2.95 | 0.00 |
| Year | 0.34 | 0.01 | 0.00 | 0.33 | 0.35 | 0.00 |
| Received support (medium) | 0.05 | 0.06 | 0.38 | -0.06 | 0.16 | 0.56 |
| Received support (high) | 0.35 | 0.12 | 0.00 | 0.12 | 0.59 | 0.01 |
| Received support (no mum and/or dad) | -0.20 | 0.07 | 0.00 | -0.33 | -0.07 | 0.01 |
| CESD total | 0.00 | 0.00 | 0.84 | -0.01 | 0.00 | 0.85 |
| Year*Received support (medium) | 0.00 | 0.01 | 0.79 | -0.01 | 0.02 | 0.82 |
| Year*Received support (high) | 0.03 | 0.02 | 0.08 | 0.00 | 0.06 | 0.16 |
| Year*Received support (no mum and/or dad) | 0.02 | 0.01 | 0.00 | 0.01 | 0.04 | 0.01 |
| **Perceived support from friends** |  |  |  |  |  |  |
| Intercept | 2.03 | 0.31 | 0.00 | 1.42 | 2.65 | 0.00 |
| Year | 0.38 | 0.02 | 0.00 | 0.35 | 0.42 | 0.00 |
| Perceived support (medium) | 0.16 | 0.15 | 0.30 | -0.14 | 0.45 | 0.49 |
| Perceived support (high) | 0.29 | 0.15 | 0.06 | -0.01 | 0.59 | 0.13 |
| CESD total | 0.00 | 0.00 | 0.99 | -0.01 | 0.01 | 0.99 |
| Year*Perceived support (medium) | -0.03 | 0.02 | 0.13 | -0.07 | 0.01 | 0.24 |
| Year*Perceived support (high) | -0.04 | 0.02 | 0.03 | -0.08 | 0.00 | 0.07 |
| **Received support from friends** |  |  |  |  |  |  |
| Intercept | 2.23 | 0.28 | 0.00 | 1.67 | 2.79 | 0.00 |
| Year | 0.36 | 0.01 | 0.00 | 0.35 | 0.37 | 0.00 |
| Received support (medium) | 0.08 | 0.06 | 0.18 | -0.04 | 0.20 | 0.32 |
| Received support (high) | 0.08 | 0.06 | 0.20 | -0.04 | 0.20 | 0.34 |
| CESD total | 0.00 | 0.00 | 0.73 | -0.01 | 0.00 | 0.78 |
| Year*Received support (medium) | -0.01 | 0.01 | 0.09 | -0.03 | 0.00 | 0.18 |
| Year*Received support (high) | -0.01 | 0.01 | 0.13 | -0.02 | 0.00 | 0.24 |
| **Positive experiences with family** |  |  |  |  |  |  |
| Intercept | 2.09 | 0.32 | 0.00 | 1.45 | 2.73 | 0.00 |
| Year | 0.33 | 0.01 | 0.00 | 0.30 | 0.36 | 0.00 |
| Positive (medium) | 0.11 | 0.13 | 0.40 | -0.14 | 0.36 | 0.58 |
| Positive (high) | 0.28 | 0.12 | 0.02 | 0.04 | 0.52 | 0.07 |
| Positive (no mum and/or dad) | -0.02 | 0.13 | 0.87 | -0.27 | 0.23 | 0.88 |
| CESD total | 0.00 | 0.00 | 0.45 | 0.00 | 0.01 | 0.59 |
| Year*Positive (medium) | 0.01 | 0.02 | 0.67 | -0.02 | 0.04 | 0.74 |
| Year*Positive (high) | 0.01 | 0.01 | 0.42 | -0.02 | 0.04 | 0.58 |
| Year*Positive (no mum and/or dad) | 0.03 | 0.02 | 0.03 | 0.00 | 0.06 | 0.09 |
| **Negative experiences with family** |  |  |  |  |  |  |
| Intercept | 2.43 | 0.30 | 0.00 | 1.84 | 3.01 | 0.00 |
| Year | 0.34 | 0.01 | 0.00 | 0.33 | 0.35 | 0.00 |
| Negative (medium) | 0.05 | 0.08 | 0.53 | -0.10 | 0.20 | 0.66 |
| Negative (high) | 0.03 | 0.08 | 0.72 | -0.12 | 0.18 | 0.78 |
| Negative (no mum and/or dad) | -0.22 | 0.07 | 0.00 | -0.35 | -0.09 | 0.00 |
| CESD total | 0.00 | 0.00 | 0.80 | -0.01 | 0.00 | 0.83 |
| Year*Negative (medium) | 0.00 | 0.01 | 0.73 | -0.02 | 0.02 | 0.78 |
| Year*Negative (high) | 0.01 | 0.01 | 0.58 | -0.01 | 0.02 | 0.70 |
| Year*Negative (no mum and/or dad) | 0.02 | 0.01 | 0.00 | 0.01 | 0.04 | 0.02 |

*Note. This table represents findings from linear mixed effect models controlling for age, gender, ethnicity, neighbourhood disadvantage, parental education and occupation, self-rated health, and depression.*

## Supplementary Index 14: Growth curve models adjusted for puberty.

| **Term** | **Estimate** | **Std error** | **P value** | **2.5 %** | **97.5 %** | **P value FDR** |
| --- | --- | --- | --- | --- | --- | --- |
| **Social contact with family** |  |  |  |  |  |  |
| Intercept | 2.33 | 0.31 | 0.00 | 1.71 | 2.95 | 0.00 |
| Year | 0.33 | 0.01 | 0.00 | 0.30 | 0.35 | 0.00 |
| Social contact (medium) | -0.02 | 0.11 | 0.84 | -0.23 | 0.19 | 0.85 |
| Social contact (high) | 0.12 | 0.11 | 0.31 | -0.11 | 0.34 | 0.48 |
| Social contact (no mum and/or dad) | -0.20 | 0.12 | 0.08 | -0.43 | 0.03 | 0.16 |
| Puberty (yes) | 0.05 | 0.06 | 0.38 | -0.06 | 0.16 | 0.56 |
| Year*Social contact (medium) | 0.01 | 0.01 | 0.34 | -0.01 | 0.04 | 0.52 |
| Year*Social contact (high) | 0.02 | 0.01 | 0.18 | -0.01 | 0.05 | 0.31 |
| Year*Social contact (no mum and/or dad) | 0.04 | 0.01 | 0.01 | 0.01 | 0.06 | 0.04 |
| **Social contact with friends** |  |  |  |  |  |  |
| Intercept | 2.21 | 0.27 | 0.00 | 1.68 | 2.74 | 0.00 |
| Year | 0.38 | 0.01 | 0.00 | 0.36 | 0.40 | 0.00 |
| Social contact (medium) | 0.07 | 0.08 | 0.40 | -0.09 | 0.23 | 0.56 |
| Social contact (high) | 0.04 | 0.08 | 0.67 | -0.13 | 0.20 | 0.72 |
| Puberty (yes) | 0.05 | 0.05 | 0.41 | -0.06 | 0.15 | 0.56 |
| Year*Social contact (medium) | -0.04 | 0.01 | 0.00 | -0.06 | -0.02 | 0.00 |
| Year*Social contact (high) | -0.04 | 0.01 | 0.00 | -0.06 | -0.02 | 0.00 |
| **Relationship status** |  |  |  |  |  |  |
| Intercept | 2.39 | 0.29 | 0.00 | 1.82 | 2.95 | 0.00 |
| Year | 0.35 | 0.01 | 0.00 | 0.34 | 0.36 | 0.00 |
| Relationship status (single) | -0.07 | 0.05 | 0.19 | -0.17 | 0.03 | 0.32 |
| Puberty (yes) | 0.04 | 0.05 | 0.48 | -0.07 | 0.15 | 0.59 |
| Year*Relationship status (single) | 0.00 | 0.01 | 0.45 | -0.02 | 0.01 | 0.57 |
| **Loneliness** |  |  |  |  |  |  |
| Intercept | 2.23 | 0.29 | 0.00 | 1.66 | 2.79 | 0.00 |
| Year | 0.35 | 0.00 | 0.00 | 0.34 | 0.36 | 0.00 |
| Loneliness (medium) | -0.15 | 0.05 | 0.01 | -0.26 | -0.05 | 0.02 |
| Loneliness (high) | -0.05 | 0.09 | 0.56 | -0.22 | 0.12 | 0.67 |
| Puberty (yes) | 0.04 | 0.06 | 0.43 | -0.07 | 0.15 | 0.56 |
| Year*Loneliness (medium) | 0.01 | 0.01 | 0.20 | -0.01 | 0.02 | 0.33 |
| Year*Loneliness (high) | 0.01 | 0.01 | 0.58 | -0.02 | 0.03 | 0.68 |
| **Perceived support from family** |  |  |  |  |  |  |
| Intercept | 2.33 | 0.33 | 0.00 | 1.67 | 2.98 | 0.00 |
| Year | 0.35 | 0.02 | 0.00 | 0.31 | 0.39 | 0.00 |
| Perceived support (medium) | -0.03 | 0.16 | 0.87 | -0.33 | 0.28 | 0.88 |
| Perceived support (high) | 0.11 | 0.16 | 0.48 | -0.20 | 0.42 | 0.59 |
| Perceived support (no mum and/or dad) | -0.16 | 0.16 | 0.31 | -0.47 | 0.15 | 0.48 |
| Puberty (yes) | 0.05 | 0.06 | 0.39 | -0.06 | 0.16 | 0.56 |
| Year*Perceived support (medium) | 0.00 | 0.02 | 0.97 | -0.04 | 0.04 | 0.97 |
| Year*Perceived support (high) | -0.01 | 0.02 | 0.71 | -0.05 | 0.03 | 0.75 |
| Year*Perceived support (no mum and/or dad) | 0.02 | 0.02 | 0.44 | -0.03 | 0.06 | 0.56 |
| **Received support from family** |  |  |  |  |  |  |
| Intercept | 2.39 | 0.30 | 0.00 | 1.80 | 2.98 | 0.00 |
| Year | 0.34 | 0.01 | 0.00 | 0.33 | 0.35 | 0.00 |
| Received support (medium) | 0.05 | 0.06 | 0.38 | -0.06 | 0.16 | 0.55 |
| Received support (high) | 0.35 | 0.12 | 0.00 | 0.12 | 0.59 | 0.01 |
| Received support (no mum and/or dad) | -0.20 | 0.07 | 0.00 | -0.33 | -0.07 | 0.01 |
| Puberty (yes) | 0.05 | 0.06 | 0.41 | -0.06 | 0.15 | 0.56 |
| Year*Received support (medium) | 0.00 | 0.01 | 0.79 | -0.01 | 0.02 | 0.82 |
| Year*Received support (high) | 0.03 | 0.02 | 0.08 | 0.00 | 0.06 | 0.15 |
| Year*Received support (no mum and/or dad) | 0.02 | 0.01 | 0.00 | 0.01 | 0.04 | 0.01 |
| **Perceived support from friends** |  |  |  |  |  |  |
| Intercept | 2.03 | 0.31 | 0.00 | 1.42 | 2.65 | 0.00 |
| Year | 0.38 | 0.02 | 0.00 | 0.35 | 0.42 | 0.00 |
| Perceived support (medium) | 0.16 | 0.15 | 0.30 | -0.14 | 0.45 | 0.48 |
| Perceived support (high) | 0.29 | 0.15 | 0.06 | -0.01 | 0.59 | 0.12 |
| Puberty (yes) | 0.04 | 0.06 | 0.44 | -0.07 | 0.15 | 0.56 |
| Year*Perceived support (medium) | -0.03 | 0.02 | 0.13 | -0.07 | 0.01 | 0.23 |
| Year*Perceived support (high) | -0.04 | 0.02 | 0.03 | -0.08 | 0.00 | 0.07 |
| **Received support from friends** |  |  |  |  |  |  |
| Intercept | 2.23 | 0.28 | 0.00 | 1.67 | 2.79 | 0.00 |
| Year | 0.36 | 0.01 | 0.00 | 0.35 | 0.37 | 0.00 |
| Received support (medium) | 0.08 | 0.06 | 0.19 | -0.04 | 0.20 | 0.32 |
| Received support (high) | 0.08 | 0.06 | 0.21 | -0.04 | 0.20 | 0.34 |
| Puberty (yes) | 0.04 | 0.06 | 0.47 | -0.07 | 0.15 | 0.59 |
| Year*Received support (medium) | -0.01 | 0.01 | 0.09 | -0.03 | 0.00 | 0.18 |
| Year*Received support (high) | -0.01 | 0.01 | 0.13 | -0.02 | 0.00 | 0.23 |
| **Positive experiences with family** |  |  |  |  |  |  |
| Intercept | 2.12 | 0.32 | 0.00 | 1.48 | 2.75 | 0.00 |
| Year | 0.33 | 0.01 | 0.00 | 0.30 | 0.36 | 0.00 |
| Positive (medium) | 0.10 | 0.13 | 0.43 | -0.15 | 0.35 | 0.56 |
| Positive (high) | 0.26 | 0.12 | 0.03 | 0.03 | 0.50 | 0.07 |
| Positive (no mum and/or dad) | -0.03 | 0.13 | 0.80 | -0.28 | 0.22 | 0.82 |
| Puberty (yes) | 0.05 | 0.06 | 0.35 | -0.06 | 0.16 | 0.53 |
| Year*Positive (medium) | 0.01 | 0.02 | 0.67 | -0.02 | 0.04 | 0.72 |
| Year*Positive (high) | 0.01 | 0.01 | 0.42 | -0.02 | 0.04 | 0.56 |
| Year*Positive (no mum and/or dad) | 0.03 | 0.02 | 0.03 | 0.00 | 0.06 | 0.08 |
| **Negative experiences with family** |  |  |  |  |  |  |
| Intercept | 2.43 | 0.30 | 0.00 | 1.84 | 3.01 | 0.00 |
| Year | 0.34 | 0.01 | 0.00 | 0.33 | 0.35 | 0.00 |
| Negative (medium) | 0.05 | 0.08 | 0.55 | -0.10 | 0.20 | 0.66 |
| Negative (high) | 0.03 | 0.08 | 0.74 | -0.13 | 0.18 | 0.77 |
| Negative (no mum and/or dad) | -0.22 | 0.07 | 0.00 | -0.36 | -0.09 | 0.00 |
| Puberty (yes) | 0.04 | 0.06 | 0.42 | -0.06 | 0.15 | 0.56 |
| Year*Negative (medium) | 0.00 | 0.01 | 0.73 | -0.02 | 0.02 | 0.76 |
| Year*Negative (high) | 0.01 | 0.01 | 0.58 | -0.01 | 0.02 | 0.68 |
| Year*Negative (no mum and/or dad) | 0.02 | 0.01 | 0.00 | 0.01 | 0.04 | 0.01 |

Note. This table represents findings from linear mixed effect models controlling for age, gender, ethnicity, baseline BMI, neighbourhood disadvantage, parental education and occupation, self-rated health and puberty. *FDR stands for false discovery rate adjustment, using the Benjamini Hochberg procedure.

## Supplementary Index 15: Growth curve models adjusted for social connection constructs.

| **Term** | **Estimate** | **Std error** | **P value** | **2.5 %** | **97.5 %** | **P value FDR** |
| --- | --- | --- | --- | --- | --- | --- |
| **Social contact with family** |  |  |  |  |  |  |
| Intercept | 2.26 | 0.39 | 0.00 | 1.50 | 3.02 | 0.00 |
| Year | 0.33 | 0.01 | 0.00 | 0.30 | 0.35 | 0.00 |
| Social contact with family (medium) | -0.05 | 0.11 | 0.63 | -0.26 | 0.16 | 0.70 |
| Social contact with family (high) | 0.06 | 0.11 | 0.63 | -0.17 | 0.28 | 0.70 |
| Social contact with family (No mum and/or dad) | -1.90 | 0.83 | 0.02 | -3.53 | -0.27 | 0.09 |
| Social contact with friends (medium) | -0.08 | 0.07 | 0.25 | -0.22 | 0.06 | 0.43 |
| Social contact with friends (high) | -0.11 | 0.07 | 0.12 | -0.26 | 0.03 | 0.26 |
| Relationship status (single) | -0.09 | 0.05 | 0.06 | -0.18 | 0.00 | 0.15 |
| Loneliness (medium) | -0.11 | 0.05 | 0.02 | -0.20 | -0.01 | 0.09 |
| Loneliness (high) | 0.00 | 0.08 | 1.00 | -0.15 | 0.15 | 1.00 |
| Perceived support from family (medium) | -0.18 | 0.15 | 0.21 | -0.47 | 0.10 | 0.38 |
| Perceived support from family (high) | -0.19 | 0.15 | 0.21 | -0.50 | 0.11 | 0.38 |
| Perceived support from family (no mum and/or dad) | 1.81 | 0.83 | 0.03 | 0.18 | 3.44 | 0.09 |
| Received support from family (medium) | 0.03 | 0.05 | 0.58 | -0.07 | 0.13 | 0.69 |
| Received support from family (high) | 0.37 | 0.11 | 0.00 | 0.15 | 0.59 | 0.01 |
| Perceived support from friends (medium) | 0.07 | 0.13 | 0.57 | -0.18 | 0.32 | 0.69 |
| Perceived support from friends (high) | 0.13 | 0.13 | 0.33 | -0.13 | 0.38 | 0.51 |
| Received support from friends (medium) | 0.03 | 0.05 | 0.53 | -0.07 | 0.14 | 0.67 |
| Received support from friends (high) | 0.03 | 0.06 | 0.65 | -0.09 | 0.14 | 0.70 |
| Positive experiences with family (medium) | 0.18 | 0.12 | 0.13 | -0.06 | 0.42 | 0.28 |
| Positive experiences with family (high) | 0.34 | 0.13 | 0.01 | 0.09 | 0.59 | 0.03 |
| Negative experiences with family (medium) | 0.07 | 0.06 | 0.30 | -0.06 | 0.19 | 0.49 |
| Negative experiences with family (high) | 0.06 | 0.07 | 0.37 | -0.07 | 0.20 | 0.52 |
| Year*Social contact with family (medium) | 0.01 | 0.01 | 0.33 | -0.01 | 0.04 | 0.51 |
| Year*Social contact with family (high) | 0.02 | 0.01 | 0.18 | -0.01 | 0.05 | 0.36 |
| Year*Social contact with family (No mum and/or dad) | 0.04 | 0.01 | 0.01 | 0.01 | 0.06 | 0.05 |
| **Social contact with friends** |  |  |  |  |  |  |
| Intercept | 2.06 | 0.38 | 0.00 | 1.32 | 2.80 | 0.00 |
| Year | 0.38 | 0.01 | 0.00 | 0.36 | 0.40 | 0.00 |
| Social contact with friends (medium) | 0.05 | 0.08 | 0.55 | -0.11 | 0.21 | 0.68 |
| Social contact with friends (high) | 0.02 | 0.09 | 0.85 | -0.15 | 0.18 | 0.89 |
| Social contact with family (medium) | -0.01 | 0.09 | 0.93 | -0.19 | 0.17 | 0.95 |
| Social contact with family (high) | 0.12 | 0.10 | 0.20 | -0.07 | 0.31 | 0.38 |
| Social contact with family (No mum and/or dad) | -1.77 | 0.83 | 0.03 | -3.40 | -0.14 | 0.09 |
| Relationship status (single) | -0.09 | 0.05 | 0.06 | -0.18 | 0.00 | 0.16 |
| Loneliness (medium) | -0.11 | 0.05 | 0.02 | -0.20 | -0.01 | 0.09 |
| Loneliness (high) | 0.00 | 0.08 | 1.00 | -0.15 | 0.15 | 1.00 |
| Perceived support from family (medium) | -0.18 | 0.15 | 0.21 | -0.47 | 0.10 | 0.38 |
| Perceived support from family (high) | -0.19 | 0.15 | 0.21 | -0.50 | 0.11 | 0.38 |
| Perceived support from family (no mum and/or dad) | 1.81 | 0.83 | 0.03 | 0.18 | 3.44 | 0.09 |
| Received support from family (medium) | 0.03 | 0.05 | 0.58 | -0.07 | 0.13 | 0.69 |
| Received support from family (high) | 0.37 | 0.11 | 0.00 | 0.15 | 0.59 | 0.01 |
| Perceived support from friends (medium) | 0.07 | 0.13 | 0.58 | -0.18 | 0.32 | 0.69 |
| Perceived support from friends (high) | 0.13 | 0.13 | 0.33 | -0.13 | 0.38 | 0.51 |
| Received support from friends (medium) | 0.03 | 0.05 | 0.53 | -0.07 | 0.14 | 0.67 |
| Received support from friends (high) | 0.03 | 0.06 | 0.65 | -0.09 | 0.14 | 0.70 |
| Positive experiences with family (medium) | 0.18 | 0.12 | 0.13 | -0.06 | 0.42 | 0.28 |
| Positive experiences with family (high) | 0.34 | 0.13 | 0.01 | 0.09 | 0.59 | 0.03 |
| Negative experiences with family (medium) | 0.07 | 0.06 | 0.30 | -0.06 | 0.19 | 0.50 |
| Negative experiences with family (high) | 0.06 | 0.07 | 0.37 | -0.07 | 0.20 | 0.52 |
| Year*Social contact with friends (medium) | -0.04 | 0.01 | 0.00 | -0.06 | -0.02 | 0.00 |
| Year*Social contact with friends (high) | -0.04 | 0.01 | 0.00 | -0.06 | -0.02 | 0.01 |
| **Relationship status** |  |  |  |  |  |  |
| Intercept | 2.19 | 0.37 | 0.00 | 1.46 | 2.91 | 0.00 |
| Year | 0.35 | 0.01 | 0.00 | 0.34 | 0.36 | 0.00 |
| Relationship status (single) | -0.07 | 0.05 | 0.18 | -0.18 | 0.03 | 0.36 |
| Social contact with family (medium) | -0.01 | 0.09 | 0.93 | -0.19 | 0.17 | 0.95 |
| Social contact with family (high) | 0.12 | 0.10 | 0.20 | -0.06 | 0.31 | 0.38 |
| Social contact with family (No mum and/or dad) | -1.76 | 0.82 | 0.03 | -3.38 | -0.15 | 0.09 |
| Social contact with friends (medium) | -0.08 | 0.07 | 0.24 | -0.22 | 0.06 | 0.42 |
| Social contact with friends (high) | -0.12 | 0.07 | 0.11 | -0.26 | 0.03 | 0.25 |
| Loneliness (medium) | -0.11 | 0.05 | 0.02 | -0.20 | -0.01 | 0.09 |
| Loneliness (high) | 0.00 | 0.08 | 1.00 | -0.15 | 0.15 | 1.00 |
| Perceived support from family (medium) | -0.18 | 0.15 | 0.21 | -0.47 | 0.10 | 0.38 |
| Perceived support from family (high) | -0.19 | 0.15 | 0.21 | -0.49 | 0.11 | 0.38 |
| Perceived support from family (no mum and/or dad) | 1.80 | 0.82 | 0.03 | 0.19 | 3.42 | 0.09 |
| Received support from family (medium) | 0.03 | 0.05 | 0.58 | -0.07 | 0.13 | 0.69 |
| Received support from family (high) | 0.37 | 0.11 | 0.00 | 0.15 | 0.59 | 0.01 |
| Perceived support from friends (medium) | 0.07 | 0.13 | 0.57 | -0.18 | 0.32 | 0.69 |
| Perceived support from friends (high) | 0.13 | 0.13 | 0.33 | -0.13 | 0.38 | 0.51 |
| Received support from friends (medium) | 0.03 | 0.05 | 0.53 | -0.07 | 0.14 | 0.67 |
| Received support from friends (high) | 0.03 | 0.06 | 0.64 | -0.09 | 0.14 | 0.70 |
| Positive experiences with family (medium) | 0.18 | 0.12 | 0.13 | -0.05 | 0.42 | 0.28 |
| Positive experiences with family (high) | 0.34 | 0.13 | 0.01 | 0.09 | 0.59 | 0.03 |
| Negative experiences with family (medium) | 0.07 | 0.06 | 0.29 | -0.06 | 0.19 | 0.49 |
| Negative experiences with family (high) | 0.06 | 0.07 | 0.37 | -0.07 | 0.20 | 0.52 |
| Year*Relationship status (single) | 0.00 | 0.01 | 0.46 | -0.02 | 0.01 | 0.59 |
| **Loneliness** |  |  |  |  |  |  |
| Intercept | 2.22 | 0.36 | 0.00 | 1.51 | 2.93 | 0.00 |
| Year | 0.35 | 0.00 | 0.00 | 0.34 | 0.36 | 0.00 |
| Loneliness (medium) | -0.14 | 0.06 | 0.01 | -0.25 | -0.03 | 0.05 |
| Loneliness (high) | -0.02 | 0.09 | 0.80 | -0.20 | 0.16 | 0.84 |
| Social contact with family (medium) | -0.01 | 0.09 | 0.93 | -0.19 | 0.17 | 0.95 |
| Social contact with family (high) | 0.12 | 0.09 | 0.20 | -0.06 | 0.31 | 0.38 |
| Social contact with family (No mum and/or dad) | -1.76 | 0.82 | 0.03 | -3.36 | -0.15 | 0.09 |
| Social contact with friends (medium) | -0.08 | 0.07 | 0.23 | -0.22 | 0.05 | 0.41 |
| Social contact with friends (high) | -0.12 | 0.07 | 0.10 | -0.26 | 0.02 | 0.24 |
| Relationship status (single) | -0.09 | 0.05 | 0.05 | -0.18 | 0.00 | 0.13 |
| Perceived support from family (medium) | -0.18 | 0.14 | 0.20 | -0.47 | 0.10 | 0.38 |
| Perceived support from family (high) | -0.19 | 0.15 | 0.20 | -0.49 | 0.10 | 0.38 |
| Perceived support from family (no mum and/or dad) | 1.80 | 0.82 | 0.03 | 0.19 | 3.41 | 0.09 |
| Received support from family (medium) | 0.03 | 0.05 | 0.57 | -0.07 | 0.13 | 0.69 |
| Received support from family (high) | 0.37 | 0.11 | 0.00 | 0.16 | 0.59 | 0.01 |
| Perceived support from friends (medium) | 0.07 | 0.12 | 0.58 | -0.17 | 0.31 | 0.69 |
| Perceived support from friends (high) | 0.12 | 0.13 | 0.34 | -0.13 | 0.37 | 0.51 |
| Received support from friends (medium) | 0.03 | 0.05 | 0.53 | -0.07 | 0.14 | 0.67 |
| Received support from friends (high) | 0.03 | 0.06 | 0.64 | -0.09 | 0.14 | 0.70 |
| Positive experiences with family (medium) | 0.18 | 0.12 | 0.13 | -0.05 | 0.42 | 0.27 |
| Positive experiences with family (high) | 0.34 | 0.13 | 0.01 | 0.10 | 0.59 | 0.03 |
| Negative experiences with family (medium) | 0.07 | 0.06 | 0.30 | -0.06 | 0.19 | 0.49 |
| Negative experiences with family (high) | 0.06 | 0.07 | 0.36 | -0.07 | 0.19 | 0.52 |
| Year*Loneliness (medium) | 0.01 | 0.01 | 0.20 | -0.01 | 0.02 | 0.38 |
| Year*Loneliness (high) | 0.01 | 0.01 | 0.58 | -0.02 | 0.03 | 0.69 |
| **Perceived support from family** |  |  |  |  |  |  |
| Intercept | 2.20 | 0.38 | 0.00 | 1.46 | 2.94 | 0.00 |
| Year | 0.35 | 0.02 | 0.00 | 0.31 | 0.39 | 0.00 |
| Perceived support from family (medium) | -0.18 | 0.17 | 0.28 | -0.51 | 0.15 | 0.47 |
| Perceived support from family (high) | -0.17 | 0.18 | 0.35 | -0.51 | 0.18 | 0.52 |
| Perceived support from family (no mum and/or dad) | 1.74 | 0.82 | 0.03 | 0.13 | 3.34 | 0.09 |
| Social contact with family (medium) | -0.01 | 0.09 | 0.92 | -0.19 | 0.17 | 0.95 |
| Social contact with family (high) | 0.12 | 0.10 | 0.20 | -0.06 | 0.31 | 0.38 |
| Social contact with family (No mum and/or dad) | -1.75 | 0.82 | 0.03 | -3.37 | -0.14 | 0.09 |
| Social contact with friends (medium) | -0.08 | 0.07 | 0.24 | -0.22 | 0.06 | 0.42 |
| Social contact with friends (high) | -0.12 | 0.07 | 0.11 | -0.26 | 0.03 | 0.25 |
| Relationship status (single) | -0.09 | 0.05 | 0.06 | -0.18 | 0.00 | 0.15 |
| Loneliness (medium) | -0.11 | 0.05 | 0.02 | -0.20 | -0.01 | 0.09 |
| Loneliness (high) | 0.00 | 0.08 | 1.00 | -0.15 | 0.15 | 1.00 |
| Received support from family (medium) | 0.03 | 0.05 | 0.58 | -0.07 | 0.13 | 0.69 |
| Received support from family (high) | 0.37 | 0.11 | 0.00 | 0.15 | 0.59 | 0.01 |
| Perceived support from friends (medium) | 0.07 | 0.13 | 0.57 | -0.18 | 0.32 | 0.69 |
| Perceived support from friends (high) | 0.13 | 0.13 | 0.33 | -0.13 | 0.38 | 0.51 |
| Received support from friends (medium) | 0.03 | 0.05 | 0.53 | -0.07 | 0.14 | 0.67 |
| Received support from friends (high) | 0.03 | 0.06 | 0.64 | -0.09 | 0.14 | 0.70 |
| Positive experiences with family (medium) | 0.18 | 0.12 | 0.13 | -0.05 | 0.42 | 0.28 |
| Positive experiences with family (high) | 0.34 | 0.13 | 0.01 | 0.09 | 0.59 | 0.03 |
| Negative experiences with family (medium) | 0.07 | 0.06 | 0.29 | -0.06 | 0.19 | 0.49 |
| Negative experiences with family (high) | 0.06 | 0.07 | 0.37 | -0.07 | 0.20 | 0.52 |
| Year*Perceived support from family (medium) | 0.00 | 0.02 | 0.98 | -0.04 | 0.04 | 1.00 |
| Year*Perceived support from family (high) | -0.01 | 0.02 | 0.71 | -0.05 | 0.03 | 0.75 |
| Year*Perceived support from family (no mum and/or dad) | 0.02 | 0.02 | 0.44 | -0.03 | 0.06 | 0.57 |
| **Received support from family** |  |  |  |  |  |  |
| Intercept | 2.21 | 0.38 | 0.00 | 1.46 | 2.97 | 0.00 |
| Year | 0.34 | 0.01 | 0.00 | 0.33 | 0.35 | 0.00 |
| Received support from family (medium) | 0.02 | 0.06 | 0.70 | -0.09 | 0.13 | 0.74 |
| Received support from family (high) | 0.27 | 0.12 | 0.02 | 0.04 | 0.51 | 0.09 |
| Received support from family (no mum and/or dad) | -1.86 | 0.83 | 0.02 | -3.48 | -0.23 | 0.09 |
| Social contact with family (medium) | -0.01 | 0.09 | 0.92 | -0.19 | 0.17 | 0.95 |
| Social contact with family (high) | 0.12 | 0.10 | 0.20 | -0.07 | 0.31 | 0.38 |
| Social contact with friends (medium) | -0.08 | 0.07 | 0.25 | -0.22 | 0.06 | 0.43 |
| Social contact with friends (high) | -0.11 | 0.07 | 0.12 | -0.26 | 0.03 | 0.26 |
| Relationship status (single) | -0.09 | 0.05 | 0.06 | -0.18 | 0.00 | 0.15 |
| Loneliness (medium) | -0.11 | 0.05 | 0.02 | -0.20 | -0.01 | 0.09 |
| Loneliness (high) | 0.00 | 0.08 | 1.00 | -0.15 | 0.15 | 1.00 |
| Perceived support from family (medium) | -0.18 | 0.15 | 0.21 | -0.47 | 0.10 | 0.38 |
| Perceived support from family (high) | -0.19 | 0.15 | 0.21 | -0.50 | 0.11 | 0.38 |
| Perceived support from family (no mum and/or dad) | 1.81 | 0.83 | 0.03 | 0.18 | 3.44 | 0.09 |
| Perceived support from friends (medium) | 0.07 | 0.13 | 0.57 | -0.18 | 0.32 | 0.69 |
| Perceived support from friends (high) | 0.13 | 0.13 | 0.33 | -0.13 | 0.38 | 0.51 |
| Received support from friends (medium) | 0.03 | 0.05 | 0.53 | -0.07 | 0.14 | 0.67 |
| Received support from friends (high) | 0.03 | 0.06 | 0.64 | -0.09 | 0.14 | 0.70 |
| Positive experiences with family (medium) | 0.18 | 0.12 | 0.13 | -0.06 | 0.42 | 0.28 |
| Positive experiences with family (high) | 0.34 | 0.13 | 0.01 | 0.09 | 0.59 | 0.03 |
| Negative experiences with family (medium) | 0.07 | 0.06 | 0.30 | -0.06 | 0.19 | 0.49 |
| Negative experiences with family (high) | 0.06 | 0.07 | 0.37 | -0.07 | 0.20 | 0.52 |
| Year*Received support from family (medium) | 0.00 | 0.01 | 0.80 | -0.01 | 0.02 | 0.84 |
| Year*Received support from family (high) | 0.03 | 0.02 | 0.07 | 0.00 | 0.06 | 0.19 |
| Year*Received support from family (no mum and/or dad) | 0.02 | 0.01 | 0.00 | 0.01 | 0.04 | 0.01 |
| **Perceived support from friends** |  |  |  |  |  |  |
| Intercept | 2.08 | 0.37 | 0.00 | 1.35 | 2.80 | 0.00 |
| Year | 0.38 | 0.02 | 0.00 | 0.35 | 0.42 | 0.00 |
| Perceived support from friends (medium) | 0.17 | 0.15 | 0.24 | -0.12 | 0.47 | 0.42 |
| Perceived support from friends (high) | 0.28 | 0.15 | 0.07 | -0.02 | 0.58 | 0.17 |
| Social contact with family (medium) | -0.01 | 0.09 | 0.93 | -0.19 | 0.17 | 0.95 |
| Social contact with family (high) | 0.12 | 0.10 | 0.20 | -0.06 | 0.31 | 0.38 |
| Social contact with family (No mum and/or dad) | -1.76 | 0.82 | 0.03 | -3.37 | -0.14 | 0.09 |
| Social contact with friends (medium) | -0.08 | 0.07 | 0.24 | -0.22 | 0.06 | 0.42 |
| Social contact with friends (high) | -0.12 | 0.07 | 0.11 | -0.26 | 0.03 | 0.25 |
| Relationship status (single) | -0.09 | 0.05 | 0.06 | -0.18 | 0.00 | 0.15 |
| Loneliness (medium) | -0.11 | 0.05 | 0.02 | -0.20 | -0.01 | 0.09 |
| Loneliness (high) | 0.00 | 0.08 | 1.00 | -0.15 | 0.15 | 1.00 |
| Perceived support from family (medium) | -0.18 | 0.15 | 0.21 | -0.47 | 0.10 | 0.38 |
| Perceived support from family (high) | -0.19 | 0.15 | 0.21 | -0.49 | 0.11 | 0.38 |
| Perceived support from family (no mum and/or dad) | 1.80 | 0.82 | 0.03 | 0.18 | 3.42 | 0.09 |
| Received support from family (medium) | 0.03 | 0.05 | 0.58 | -0.07 | 0.13 | 0.69 |
| Received support from family (high) | 0.37 | 0.11 | 0.00 | 0.15 | 0.59 | 0.01 |
| Received support from friends (medium) | 0.03 | 0.05 | 0.53 | -0.07 | 0.14 | 0.67 |
| Received support from friends (high) | 0.03 | 0.06 | 0.64 | -0.09 | 0.14 | 0.70 |
| Positive experiences with family (medium) | 0.18 | 0.12 | 0.13 | -0.05 | 0.42 | 0.28 |
| Positive experiences with family (high) | 0.34 | 0.13 | 0.01 | 0.09 | 0.59 | 0.03 |
| Negative experiences with family (medium) | 0.07 | 0.06 | 0.29 | -0.06 | 0.19 | 0.49 |
| Negative experiences with family (high) | 0.06 | 0.07 | 0.37 | -0.07 | 0.19 | 0.52 |
| Year*Perceived support from friends (medium) | -0.03 | 0.02 | 0.14 | -0.07 | 0.01 | 0.28 |
| Year*Perceived support from friends (high) | -0.04 | 0.02 | 0.03 | -0.08 | 0.00 | 0.09 |
| **Received support from friends** |  |  |  |  |  |  |
| Intercept | 2.18 | 0.36 | 0.00 | 1.47 | 2.89 | 0.00 |
| Year | 0.36 | 0.01 | 0.00 | 0.35 | 0.37 | 0.00 |
| Received support from friends (medium) | 0.08 | 0.06 | 0.20 | -0.04 | 0.20 | 0.38 |
| Received support from friends (high) | 0.07 | 0.06 | 0.31 | -0.06 | 0.19 | 0.51 |
| Social contact with family (medium) | -0.01 | 0.09 | 0.92 | -0.19 | 0.17 | 0.95 |
| Social contact with family (high) | 0.12 | 0.09 | 0.20 | -0.06 | 0.31 | 0.38 |
| Social contact with family (No mum and/or dad) | -1.76 | 0.82 | 0.03 | -3.36 | -0.16 | 0.09 |
| Social contact with friends (medium) | -0.08 | 0.07 | 0.23 | -0.22 | 0.05 | 0.41 |
| Social contact with friends (high) | -0.12 | 0.07 | 0.10 | -0.26 | 0.02 | 0.24 |
| Relationship status (single) | -0.09 | 0.05 | 0.05 | -0.18 | 0.00 | 0.13 |
| Loneliness (medium) | -0.11 | 0.05 | 0.02 | -0.20 | -0.01 | 0.09 |
| Loneliness (high) | 0.00 | 0.08 | 1.00 | -0.15 | 0.15 | 1.00 |
| Perceived support from family (medium) | -0.18 | 0.14 | 0.20 | -0.47 | 0.10 | 0.38 |
| Perceived support from family (high) | -0.19 | 0.15 | 0.20 | -0.49 | 0.10 | 0.38 |
| Perceived support from family (no mum and/or dad) | 1.80 | 0.82 | 0.03 | 0.20 | 3.40 | 0.09 |
| Received support from family (medium) | 0.03 | 0.05 | 0.58 | -0.07 | 0.13 | 0.69 |
| Received support from family (high) | 0.37 | 0.11 | 0.00 | 0.16 | 0.59 | 0.01 |
| Perceived support from friends (medium) | 0.07 | 0.12 | 0.57 | -0.17 | 0.31 | 0.69 |
| Perceived support from friends (high) | 0.12 | 0.13 | 0.34 | -0.13 | 0.37 | 0.51 |
| Positive experiences with family (medium) | 0.18 | 0.12 | 0.13 | -0.05 | 0.42 | 0.27 |
| Positive experiences with family (high) | 0.34 | 0.13 | 0.01 | 0.10 | 0.59 | 0.03 |
| Negative experiences with family (medium) | 0.07 | 0.06 | 0.29 | -0.06 | 0.19 | 0.49 |
| Negative experiences with family (high) | 0.06 | 0.07 | 0.36 | -0.07 | 0.19 | 0.52 |
| Year*Received support from friends (medium) | -0.01 | 0.01 | 0.09 | -0.03 | 0.00 | 0.23 |
| Year*Received support from friends (high) | -0.01 | 0.01 | 0.13 | -0.02 | 0.00 | 0.28 |
| **Positive experiences with family** |  |  |  |  |  |  |
| Intercept | 2.24 | 0.39 | 0.00 | 1.47 | 3.02 | 0.00 |
| Year | 0.33 | 0.01 | 0.00 | 0.30 | 0.36 | 0.00 |
| Positive experiences with family (medium) | 0.16 | 0.14 | 0.25 | -0.11 | 0.42 | 0.43 |
| Positive experiences with family (high) | 0.30 | 0.14 | 0.03 | 0.02 | 0.57 | 0.09 |
| Positive experiences with family (no mum and/or dad) | -1.89 | 0.83 | 0.02 | -3.52 | -0.25 | 0.09 |
| Social contact with family (medium) | -0.01 | 0.09 | 0.92 | -0.19 | 0.17 | 0.95 |
| Social contact with family (high) | 0.12 | 0.10 | 0.21 | -0.07 | 0.31 | 0.38 |
| Social contact with friends (medium) | -0.08 | 0.07 | 0.25 | -0.22 | 0.06 | 0.43 |
| Social contact with friends (high) | -0.11 | 0.07 | 0.12 | -0.26 | 0.03 | 0.26 |
| Relationship status (single) | -0.09 | 0.05 | 0.06 | -0.18 | 0.00 | 0.16 |
| Loneliness (medium) | -0.11 | 0.05 | 0.02 | -0.20 | -0.01 | 0.09 |
| Loneliness (high) | 0.00 | 0.08 | 1.00 | -0.15 | 0.15 | 1.00 |
| Perceived support from family (medium) | -0.18 | 0.15 | 0.21 | -0.47 | 0.10 | 0.38 |
| Perceived support from family (high) | -0.19 | 0.15 | 0.21 | -0.50 | 0.11 | 0.38 |
| Perceived support from family (no mum and/or dad) | 1.82 | 0.83 | 0.03 | 0.18 | 3.45 | 0.09 |
| Received support from family (medium) | 0.03 | 0.05 | 0.58 | -0.07 | 0.13 | 0.69 |
| Received support from family (high) | 0.37 | 0.11 | 0.00 | 0.15 | 0.59 | 0.01 |
| Perceived support from friends (medium) | 0.07 | 0.13 | 0.57 | -0.18 | 0.32 | 0.69 |
| Perceived support from friends (high) | 0.13 | 0.13 | 0.33 | -0.13 | 0.38 | 0.51 |
| Received support from friends (medium) | 0.03 | 0.05 | 0.53 | -0.07 | 0.14 | 0.67 |
| Received support from friends (high) | 0.03 | 0.06 | 0.65 | -0.09 | 0.14 | 0.70 |
| Negative experiences with family (medium) | 0.07 | 0.06 | 0.30 | -0.06 | 0.19 | 0.49 |
| Negative experiences with family (high) | 0.06 | 0.07 | 0.37 | -0.07 | 0.20 | 0.52 |
| Year*Positive experiences with family (medium) | 0.01 | 0.02 | 0.67 | -0.02 | 0.04 | 0.71 |
| Year*Positive experiences with family (high) | 0.01 | 0.01 | 0.42 | -0.02 | 0.04 | 0.56 |
| Year*Positive experiences with family (no mum and/or dad) | 0.03 | 0.02 | 0.03 | 0.00 | 0.06 | 0.09 |
| **Negative experiences with family** |  |  |  |  |  |  |
| Intercept | 2.21 | 0.39 | 0.00 | 1.45 | 2.97 | 0.00 |
| Year | 0.34 | 0.01 | 0.00 | 0.33 | 0.35 | 0.00 |
| Negative experiences with family (medium) | 0.08 | 0.08 | 0.31 | -0.07 | 0.23 | 0.50 |
| Negative experiences with family (high) | 0.04 | 0.08 | 0.59 | -0.11 | 0.20 | 0.70 |
| Negative experiences with family (no mum and/or dad) | -1.85 | 0.83 | 0.03 | -3.48 | -0.23 | 0.09 |
| Social contact with family (medium) | -0.01 | 0.09 | 0.92 | -0.19 | 0.17 | 0.95 |
| Social contact with family (high) | 0.12 | 0.10 | 0.21 | -0.07 | 0.31 | 0.38 |
| Social contact with friends (medium) | -0.08 | 0.07 | 0.25 | -0.22 | 0.06 | 0.43 |
| Social contact with friends (high) | -0.11 | 0.07 | 0.12 | -0.26 | 0.03 | 0.26 |
| Relationship status (single) | -0.09 | 0.05 | 0.06 | -0.18 | 0.00 | 0.16 |
| Loneliness (medium) | -0.11 | 0.05 | 0.02 | -0.20 | -0.01 | 0.09 |
| Loneliness (high) | 0.00 | 0.08 | 1.00 | -0.15 | 0.15 | 1.00 |
| Perceived support from family (medium) | -0.18 | 0.15 | 0.21 | -0.47 | 0.10 | 0.38 |
| Perceived support from family (high) | -0.19 | 0.15 | 0.21 | -0.50 | 0.11 | 0.38 |
| Perceived support from family (no mum and/or dad) | 1.81 | 0.83 | 0.03 | 0.18 | 3.44 | 0.09 |
| Received support from family (medium) | 0.03 | 0.05 | 0.58 | -0.07 | 0.13 | 0.69 |
| Received support from family (high) | 0.37 | 0.11 | 0.00 | 0.15 | 0.59 | 0.01 |
| Perceived support from friends (medium) | 0.07 | 0.13 | 0.57 | -0.18 | 0.32 | 0.69 |
| Perceived support from friends (high) | 0.13 | 0.13 | 0.33 | -0.13 | 0.38 | 0.51 |
| Received support from friends (medium) | 0.03 | 0.05 | 0.53 | -0.07 | 0.14 | 0.67 |
| Received support from friends (high) | 0.03 | 0.06 | 0.64 | -0.09 | 0.14 | 0.70 |
| Positive experiences with family (medium) | 0.18 | 0.12 | 0.13 | -0.06 | 0.42 | 0.28 |
| Positive experiences with family (high) | 0.34 | 0.13 | 0.01 | 0.09 | 0.59 | 0.03 |
| Year*Negative experiences with family (medium) | 0.00 | 0.01 | 0.73 | -0.02 | 0.02 | 0.76 |
| Year*Negative experiences with family (high) | 0.01 | 0.01 | 0.57 | -0.01 | 0.02 | 0.69 |
| Year*Negative experiences with family (no mum and/or dad) | 0.02 | 0.01 | 0.00 | 0.01 | 0.04 | 0.02 |
